# Free/low-cost physical activity opportunities for children and adolescents: Parental perspectives on participation, access and provision

**DOI:** 10.1371/journal.pgph.0005770

**Published:** 2026-11-03

**Authors:** Roisin McCafferty, Angela Carlin, Róisín Carney, Marie H. Murphy, Alison M. Gallagher, Ciara Fitzpatrick, S. Maria O’Kane

**Affiliations:** 1 Institute of Nursing and Health Research, Ulster University, Derry/Londonderry, United Kingdom; 2 Centre for Exercise Medicine, PA and Health, Sports and Exercise Sciences Research Institute, Ulster University, Belfast, United Kingdom; 3 Physical Activity for Health Research Centre (PAHRC), Institute for Sport, Physical Education and Health Sciences, University of Edinburgh, Edinburgh, United Kingdom; 4 Nutrition Innovation Centre for Food and Health (NICHE), Biomedical Sciences Research Institute, Ulster University, Coleraine, United Kingdom; 5 School of Law, Ulster University, Belfast, United Kingdom; Universiti Malaya, MALAYSIA

## Abstract

Physical activity (PA) in childhood and adolescence has established health benefits. Despite this many children worldwide do not meet PA guidelines, with implications for future health. Cost is a recognised barrier to participation and may exacerbate socioeconomic inequalities, particularly during the ongoing cost-of-living crisis. Free/low-cost PA opportunities may help promote equitable access to PA for children, but less is known about how families experience and access these opportunities and the factors influencing engagement. This study explored parental perspectives on children’s participation in free/low-cost PA opportunities, local provision, and future needs. Differences in participation by indicators of socioeconomic status (SES) were also examined. A cross-sectional questionnaire (n = 855) and semi-structured interviews (n = 29) were conducted with parents/guardians of children aged 4–16 years in Northern Ireland. Quantitative data were analysed descriptively and using between-group comparisons, while qualitative data were analysed thematically. Almost all parents/guardians (99%) reported weekly participation in at least one free/low-cost PA opportunity, most commonly informal activities such as walking and outdoor play. However, 76% perceived insufficient local opportunities, and only 10% considered provision adequate. Since the cost-of-living crisis began in 2021, 29% reported increased engagement in free activities, including walking and running. Qualitative findings highlighted barriers beyond cost, including limited awareness, location, transport, and the quality and suitability of provision. Although free/low-cost PA opportunities were widely used, parents identified gaps in local provision and access. Participation did not differ across most SES indicators, but this does not necessarily indicate equitable access, particularly for adolescents, girls, and children with additional needs. Findings suggest that removing cost barriers alone may be insufficient as financial, environmental, social and individual factors influence access and engagement with free/low cost opportunities. A coordinated approach encompassing increased availability, improved facilities, better promotion, and targeted support for low-income families is needed to ensure equitable opportunities for children’s PA.

## Introduction

Physical activity (PA) during childhood and adolescence is associated with numerous short-term health benefits on cognition, academic performance, and mental health [1,2] while longer-term benefits relate to a reduced risk of obesity and depression and improved muscle, bone and cardiometabolic health [1,3]. Despite recommendations from the World Health Organisation (WHO) that children and adolescents should engage in at least 60 minutes of moderate-to-vigorous PA (MVPA) daily [4], globally participation remains low with 81% of adolescents (11–17 years) not meeting these guidelines [5]. In Northern Ireland (NI), adherence to the guidelines is similarly low with only 21% of primary school children (10–12 years) and 16% of post-primary pupils (12–18 years) meeting current PA guidelines [6]. Some commonly cited barriers to PA among children and adolescents include a lack of fun, motivation, perceived lack of competence, body image, negative experiences, limited environmental opportunities and lack of support from family and friends [7–9]. Cost has emerged as a significant constraint, especially in the context of the ongoing cost-of-living crisis. Evidence suggests that families have reduced spending on children’s sport and PA since the cost-of-living crisis began in late 2021 [10]. In addition, 30% of parents/guardians were unable to afford the equipment and clothing that their children needed to participate in sport or PA and since 2021, 24% of parents/guardians had used credit or borrowed money to cover sport and PA-related costs for their children [10]. These pressures contribute to a persistent ‘socioeconomic participation gap’ whereby children from families with higher socioeconomic status (SES) are reported to be more physically active and participate in more sport than those with lower SES [6,11,12].

In this context, access to high-quality free/low-cost PA opportunities is increasingly important to support participation and equitable access for all children and families. Structured activities such as ‘Parkrun’, alongside more informal PA resources or opportunities including playparks, green spaces, recreational facilities and walkways have been associated with higher levels of PA [13,14]. However, access to such opportunities is often unequally distributed with disadvantaged communities having reduced availability of safe and accessible play spaces [15]. This is compounded by declining public investment in play provision for children across the United Kingdom (UK) [16,17]. Access to PA opportunities is influenced by a range of factors operating across multiple and interacting levels. The Health Equity Framework provides a useful lens for understanding these influences, placing equity and fair access to resources and opportunities at the centre of health and wellbeing and recognising the interaction between individual, social, environmental and structural factors [18].

While emerging evidence suggests that removing financial barriers can increase PA participation and may help to reduce inequalities, findings are mixed, particularly for children and adolescents [19]. A recent scoping review concluded that while subsidised or free opportunities can improve participation and engage children from lower socioeconomic backgrounds, the impact of these initiatives decreases with increasing deprivation [20]. Research suggests that free/low-cost activities (i.e., those with no participation fee/membership or specialised equipment required) alone may not encourage those living in disadvantaged neighbourhoods to take up regular PA [21]. This suggests that additional factors may influence whether children are able to access and benefit from free or low-cost provision. However, existing research has largely focused on whether free or subsidised opportunities increase participation, with less attention given to how families experience these opportunities and the factors that may facilitate or constrain access. Understanding these factors is important for informing strategies to promote equitable PA participation. To address this gap, the aim of this mixed-methods study was to explore parental perspectives on children’s participation in free/low-cost PA opportunities, factors affecting participation, local availability and views on future provision. Differences in children’s participation based on indicators of socioeconomic status (SES) were also examined.

## Materials and methods

### Study design

This mixed-methods study incorporated a cross-sectional questionnaire and online/telephone interviews. The inclusion criteria were those who self-identified as being parents/guardians (aged >18 years) of a child or children aged between 4 and 16 years and living in NI. Ethical approval was obtained from Ulster University Nursing and Health Research Ethics Filter Committee (FCNUR-23–091). All participants were asked to read the Participant Information Sheet before providing informed consent electronically.

### Questionnaire

This study formed part of a larger research project, with full details of the questionnaire methodology published previously [10]. In brief, a cross-sectional online questionnaire (Jisc; Bristol, UK) was administered between 11^th^ March and 29^th^ May 2024. Based on census data from 2021 that reported there were 1,098,200 adults aged 20–64 years living in NI, an a priori sample size calculation was performed. This indicated that a minimum target of 663 participants (99%, α = 0.05) was required. This was used to ensure an adequate sample size was recruited, rather than to achieve population representativeness. Participants were recruited over a twelve-week period from March–June 2024, predominantly via social media (Facebook, X and Instagram) using a non-probability, snowball sampling approach, whereby participants shared the study with friends and family. Parent groups, sports clubs, community organisations and public services (e.g., libraries) were also contacted to disseminate the link to the questionnaire. As a non-probability sampling approach was used, the sample may not be representative of the wider population and findings should be interpreted with consideration of potential selection bias. No incentives were provided for participation.

Eligibility criteria included being a parent or guardian aged ≥18 years of at least one child aged 4–16 years living in Northern Ireland (NI). The term ‘parent/guardian’ was intended to include any adult with a caregiving role for the child. Screening questions were used to confirm eligibility prior to participation, and all respondents provided informed consent electronically. The survey was designed to take approximately 10 minutes to complete, and only fully completed responses were recorded on the Jisc platform.

The questionnaire consisted of five sections: 1) demographic information and SES; 2) parental support for PA; 3) impact of the cost-of-living crisis; 4) impact of the cost-of-living crisis on access to sport and PA opportunities; 5) no/low- cost PA opportunities. The present study focuses on data from Section 5.

Parents/guardians were asked to self-report their gender and age. Based on the methodology of Donnelly et al. [22], several variables were collected to determine SES. Participants were asked to provide their home postcode which was entered into the NI Multiple Deprivation Measures [23] website and the deprivation score was recorded. Deprivation scores ranged from 0 to 890 and were divided into tertiles (Most deprived, 0–296; Average, 297–593; Least deprived, 594–890). From the NI Multiple Deprivation Measures system [23], local government area and urban, rural or mixed urban/rural classifications were extracted. Data was also gathered on household income (before tax and deductions) [24], parents’ occupations and the highest level of education attained by each parent. The median annual salary for full time employees in NI was £30,000 in 2022, which was used to categorise high and low household income. Information was also collected on benefit entitlement. Participants were asked to indicate the number of children aged 4–16 years that lived at home with them who they have regular responsibility for. Participants were also asked to indicate if they share the care of their children with a partner, ex-partner, grandparent or if they were a single parent [25].

Parental support for PA was assessed using the Activity Support Scale for Multiple Groups (ACTS-MG) survey [26]. Participants were asked to indicate how often their children take part in any free/low-cost PA opportunities. Participation in free/low-cost PA opportunities was categorised based on the Children's Sport Participation and Physical Activity (CSPPA) Study methodology [6] into two groups, 1) Participation in a free/low-cost PA opportunity at least once a week (options included: daily; 4–5 times per week; 2–3 times per week; once a week); or 2) Less frequent participation in free/low-cost PA opportunities (options included once or twice a month; once or twice a year; never). Activities were defined as free/low-cost PA opportunities if no participation fee/membership or specialised equipment was required to participate. Options included within this question were walking, cycling, jogging, running, exercising at home, outdoor play park and outdoor gym.

To assess perceived access to free/low-cost PA opportunities, parents/guardians were asked *“Do you think there are enough free or low-cost PA opportunities available for children and young people?”.* Parents/guardians were also asked to specify the types of free/low-cost PA opportunities available for children and young people in their local area. In addition, parents/guardians were asked to indicate *“What types of free or low-cost PA opportunities would you like to see in your local area for children and young people?”*

### Interviews

At the end of the questionnaire, participants were advised that there was an opportunity to take part in a follow up interview (lasting no longer than 30 minutes). Those interested (n = 87) were asked to provide their contact details on a separate expression of interest form (MS Forms) where their data could not be linked to the anonymous online questionnaire. To support representative sampling, interested participants provided their gender and home postcode when submitting their expression of interest, enabling deprivation scores to be retrieved [23]. Deprivation scores ranged from 0 to 890 and were divided into tertiles (Most deprived, 0–296; Average, 297–593; Least deprived, 594–890). The sample size (n = 29) was considered sufficient to produce in-depth, rich accounts of lived experiences and to enable analytic generalisation, meaning the findings may be transferable to similar contexts rather than statistically representative of the broader population [27]. The sample demonstrated balanced representation across deprivation tertiles, and there was a sample from across the eleven council areas in NI. Invitations were sent to those interested in taking part in the interviews and informed consent was obtained electronically using Jisc (Bristol, UK).

Given the exploratory nature of the study, qualitative data were used to provide in-depth understanding of issues identified in the questionnaire. Semi-structured interviews were conducted via Microsoft Teams (Redmond, United States) or telephone from 17^th^ May to 30^th^ September 2024. The topic guide (S1 Table), informed by existing literature and findings of the online questionnaire [10], comprised three sections: 1) Background information; 2) Impact of the cost-of-living crisis; and 3) Free or low-cost PA opportunities. This paper presents data from Section 3, with findings from Sections 1 and 2 presented elsewhere. The topic guide was used to ensure consistency across interviews while allowing flexibility for participants to elaborate on their experiences.

Interviews began with brief demographic questions (e.g., employment status, annual household income, the ages of their children, and caregiving arrangements) followed by open-ended questions and prompts to explore responses in greater depth for example, “Why do you think this might be?”, “Can you tell us a little more about this?”. The guide was developed in consultation with the research team and piloted with two parents/guardians to test the construct validity, clarity and flow. Minor refinements to wording and prompts were made prior to data collection; these pilot interviews are not included in the study results. At the beginning of the interview and following an overview of the interview process explaining that the discussion would focus on the participants’ experiences and thoughts, participants were asked to provide verbal consent. All interviews were recorded. For those conducted via telephone, a digital recorder (Sony ICD-PX370; Sony Group Corporation, Tokyo, Japan) was used while the record functionality was used for interviews conducted via Microsoft Teams. Most interviews were conducted via Microsoft Teams (n = 21; 72%), with a mean interview duration of 29 minutes (Standard Deviation (SD) 9).

### Data analysis

#### Quantitative (Questionnaire).

Data were analysed using SPSS v29 (IBM Corporation, New York, USA). Descriptive statistics are presented as means (SD) for numerical data and counts (%) for categorical data. Given the exploratory nature of the questionnaire and the non-probability sampling approach, analyses were primarily descriptive.

Data distributions were assessed for normality using the Shapiro–Wilk test alongside visual inspection of histograms and Q–Q plots. As several continuous variables were non-normally distributed, non-parametric tests were used for group comparisons. The Mann-Whitney U test was used for comparisons between two groups (for example household income; low vs high). The Kruskal Wallis test was used to compare between 3 or more means (for example index of multiple deprivation tertiles). For inferential analyses, categorical variables were assessed using Chi-square tests, with Fisher’s exact test applied where appropriate. To ensure statistical robustness, analyses were restricted to variables with adequate cell counts. Where any subgroup contained fewer than five observations, it was not included in the analysis. Statistical significance was set at p < .05; however, given the exploratory design and non-probability sampling approach, these findings should be interpreted as indicative of patterns rather than of definitive group differences.

#### Qualitative (Questionnaire).

Components of the online questionnaire had built-in, optional answer probes so that further information could be obtained to contextualise data on participants experiences. Responses to open-ended questions were analysed thematically using NVivo v14 (QSR International Pty Ltd, Doncaster, VIC, Australia) based on the Braun and Clarke methodology [28]. A six-phase deductive approach was used: (1) data familiarization, (2) generating initial codes, (3) searching for themes, (4) reviewing themes, (5) defining and naming themes, and (6) producing the report. Initially, each meaningful quote or key example was assigned a code. Codes were then grouped together to develop themes that were representative of the coded excerpts. Themes were agreed by three authors (AC, RMC, SMO’K). Once coding had been completed and checked for all open-ended questions, definitions and names were then assigned to each theme. Illustrative quotes highlight key themes identified within the questionnaire.

#### Qualitative (Interviews).

Following familiarisation with the data (i.e., listening to the audio-recordings and rereading transcripts), interviews were transcribed verbatim. Transcripts were uploaded to NVivo (version 14; QSR International Pty Ltd, Doncaster, VIC, Australia) which allowed data to be organised and coded. The analysis was researcher driven and transcripts were analysed using reflexive thematic analysis, following the Braun & Clarke six-phase approach [29]. Coding was conducted by three members of the research team (SMO’K, RMcC, and RC), who engaged in ongoing discussions to refine interpretations. To support reflexive practice, the research team remained cognisant of their own positionality throughout the study, including their roles as parents, their involvement in coaching or leadership within sports clubs, and their professional backgrounds as PA researchers.

Initially, data were coded for patterns of meaning relevant to the research aim. Although the topic schedule was informed by the questionnaire findings, coding was conducted without applying a pre-existing framework, allowing themes to emerge from participant interviews. While this initial coding was rich and detailed (n = 90 codes specifically for the questions related to free/low-cost PA opportunities), it helped to avoid under-analysis of the data [25,28,30]. As the analysis progressed, codes were clustered and interpreted to generate broader themes that captured shared patterns across the dataset. Coding and theme development were discussed collaboratively within the research team to ensure reflexive engagement with the data. Codes were reviewed for patterns and grouped to form sub-themes that reflected the underlying meaning of the coded data. Overarching themes were developed and agreed upon by the research through iterative discussion and adjustment. Quotes from parents/guardians were used to highlight key themes identified during the interviews. The interviews were conducted in accordance with the Consolidated Criteria for Reporting Qualitative Research (COREQ) checklist [31].

The Health Equity Framework [18] was used as a conceptual lens to interpret the findings.

## Results

### Questionnaire

#### Demographic characteristics.

In total, n = 855 participants completed the questionnaire, exceeding the initial sample size calculation. Of these, 65% (n557) participants provided free-text responses to at least one of three open-ended questions within the ‘free/low-cost PA opportunities’ section of the questionnaire. The responses from the n = 855 parents/guardians related to n = 1957 children (50.5% male, mean age 10.1 (SD 3.8) years). The mean number of children per participant was 2 (SD 0.9) with representation from all eleven Local Council areas in NI.

As outlined in Table 1, the majority of participants resided in rural areas (49.4%). Among those in employment, the largest proportion were in Professional Occupations (32.8%). In terms of benefit entitlement, 28.7% of participants or someone in their household reported receiving benefits such as Universal Credit, Personal Independence Payment (PIP), Child Disability Living Allowance (DLA), Carer’s Allowance or Attendance Allowance.

**Table 1:** Participant characteristics of questionnaire respondents (n = 855) and interview participants (n = 29).

	Questionnaire		Interviews	
	n	%	n	%
**Gender** Female Male / Other / Prefer not to say	784 71	91.6 8.4	24 5	82.8 17.2
**Age (years)** ≤30 31-50 ≥50	29 769 57	3.4 89.9 6.6	– 24 5	– 82.8 17.2
**NI Multiple Deprivation Measure** Tertile 1 (0–296, most deprived) Tertile 2 (297–593, average) Tertile 3 (594–890, least deprived)	219 358 247	25.6 41.8 28.9	10 9 10	34.5 31.0 34.5
**Place of residence** Urban Rural Mixed urban/rural	331 423 70	38.7 49.4 8.2	15 12 2	51.7 41.4 6.9
**Number of children** 1 2 ≥3	176 382 297	20.6 44.6 34.7	7 11 11	24.1 37.9 37.9
**Education (highest level completed)** Secondary or below Further education Higher education	82 162 611	9.6 18.9 71.5	– 3 26	– 10.3 89.7
**Employment status** Not employed (including student / carer / retired) Employed / self-employed Multiple job holding	84 717 54	9.8 83.8 6.3	– 29 –	– 100 –
Responsibility for children* Shared with partner/ex-partner/grandparent(s) Single parent Other	784 117 14	85.7 12.8 1.5	28 – –	96.7 – –
**Household income** Low (<£29,999 per annum) High (>£30,000 per annum) Prefer not to say	185 617 53	21.6 72.1 6.2	5 24 –	17.2 82.8 –

Individual postcodes were searched on NI Multiple Deprivation Measures 2017 Lookup Tool and the 890 super output areas were ranked from Tertile 1 (0–296; most deprived), Tertile 2 (297–593, average) and Tertile 3 (594–890; least deprived)

*Multi-answer question

Categories with small cell counts (<5) were combined or suppressed where necessary to protect participant confidentiality; remaining small cells are presented where risk of identification is considered minimal.

#### Participation in free/low-cost PA opportunities.

Almost all parents/guardians (99.3%) indicated that their children participated in at least one free/low-cost PA opportunity at least once per week. The most commonly reported activities were walking (91.3%) or attending an outdoor playpark (63.7%). Approximately half of parents/guardians reported that their children were exercising at home (57.7%), cycling (51.9%) or going to council or community pitches (52.3%). Outdoor gyms were reported to be used infrequently by parents/guardians with only 9.8% of parents/guardians reporting that their children used these facilities at least once per week.

Overall 40.8% reported that their children took part in up to three free/low-cost PA opportunities at least once per week (below group average),19.6% reported their children participated in four activities (at group average), and 39.5% reported their children participated in more than five or more free/low-cost PA opportunities at least once per week (above average). Children who participated more frequently (i.e., > 4 activities per week) were reported to have higher levels of parental support for PA (*P*=<0.001) and greater access to sports equipment at home (*P*=<0.001); however, these findings should be interpreted with caution given the exploratory nature of the study.

As outlined in S2 Table, no statistically significant differences in participation in free/low-cost PA opportunities were observed across most indicators of SES including household income, index of multiple deprivation, benefit entitlement, employment status or between those residing in urban, rural or mixed urban/rural areas (*P* > 0.05). However, participation differed significantly by education level (*P* = 0.024), although there was no clear socioeconomic gradient.

#### Perceived access to free/low-cost PA opportunities.

More than three quarters of parents/guardians (76.3%) felt that there were not enough free/low-cost PA opportunities available for children and young people. A further 13.5% were unsure, while 10.3% thought there were enough free/low-cost PA opportunities available. No statistically significant differences in perceived access to free/low-cost PA opportunities were detected based on parental support for PA (*P =* 0.206) (S3 Table). There was evidence of differences in perceived access based on benefit entitlement (*P*=<0.001) (S4 Table). However, across other indicators of socioeconomic status, including household income, index of multiple deprivation, family structure, and employment status, no statistically significant differences were observed. Similarly, no statistically significant differences in perceived access were detected between those residing in urban, rural, or mixed urban/rural localities (*P* = 0.849). As with participation outcomes, these findings should be interpreted with caution and considered exploratory, reflecting patterns within the sample rather than conclusive population-level differences.

#### Qualitative responses (questionnaire).

The main themes to emerge from the open-ended questions in relation to free/low-cost PA opportunities for children and adolescents were: 1) Current provision of free/low-cost PA opportunities; 2) Future provision of free/low-cost PA opportunities; and 3) Gaps and barriers in provision (Table 2).

**Table 2:** Thematic analysis of open-ended responses to ‘free/low-cost PA opportunities’ survey questions.

Overall Theme	Sub-theme	Description	Quotes
Current provision of free/low-cost PA opportunities	Sports Clubs	Sports clubs provided structured opportunities, although costs varied across different sports and organisations.	“GAA activities are all free, bar a small membership fee on an annual basis (£40 per family).” *(ID 179: female, 41-50y, 4 children, employed part-time)*
			“My children attend GAA sport which is low cost in relative terms compared to other organised sport/physical activities, e.g., gymnastics/swimming.” (*ID 356: female, 41-50y, 1 child, employed full-time)*
	Running or walking	Informal and community-based activities were perceived as accessible and low-cost opportunities for regular participation.	“Junior parkrun and parkrun are great!” *(ID 276: female, 31-40y, 3 children, employed full-time)*
			“Our local running club is free and our kids do Parkrun and Junior Parkrun each week too*.” (ID 641*: *female, 41-50y, 2 children, employed full-time)*
	Access to nature	Natural environments were widely valued as free and flexible spaces for physical activity.	“Beach - lots of space to run about, swim, paddleboard etc.” *(ID 313: female, 41-50y, 2 children, employed part-time)*
			“Local woods and forest parks that provide an element of climbing/rambling and having adventures.” *(ID 531: female, 41-50y, 1 child, employed full-time)*
	Activities at home	Home-based activities were used as accessible alternatives where external opportunities were limited.	“Skipping, playing basketball & netball outside as we have a basketball basket and a net [at] home.” (*ID 203: female, 41-50y, 2 children, unemployed)*
			“Play at home is the main PA my children access, we are lucky to have been able to get various sports equipment and have the space for our children to use them.” *(ID 356: female, 31-40y, 2 child, employed part-time)*
	Facilities	Public facilities such as parks, and swimming pools were identified as important community resources for free/low-cost activities.	“Swimming pool costs are quite low for children.” *(ID 629: female, 31-40y, 2 children, employed full-time)*
			“There are a lot of play parks and areas of outstanding beauty to avail [of].” *(ID 635: female, 41-50y, 2 children, employed part-time)*
Future provision of free/low-cost PA opportunities	Access to local facilities	Participants emphasised the need for more accessible, well-equipped local facilities within walking distance.	“Indoor, spaces for families, children and young people like that which Belfast City Council have [at] 2 Royal Avenue [community and cultural hub]. Would be a lovely and beneficial community space and dry and warm with dancing, music, arts and play facility.” *(ID 275: female, 41-50y, 2 children, employed full-time)*
			“A park area with football pitches, basketball court that can be accessed for free.” *(ID 388: female, 31-40y, 2 children, employed part-time)*
	Swimming	Swimming was identified as an important but often deemed a more costly activity requiring greater affordability and access.	“Swimming (they are very expensive & I think this should be priority, as this is a key life skill).” *(ID 177: female, 41-50y, 2 children, employed part-time)*
			“It would be good if the council could offer cheaper swimming.” *(ID 86: female, 41-50y, 2 children, employed part-time)*
	Summer schemes / camps	Demand for affordable, accessible holiday programmes exceeded current availability.	“Holiday camps and other activities to accept childcare vouchers. More camps offered by councils. Affordable but not enough places to meet demand.” *(ID 223: female, 41-50y, 2 children, self-employed)*
			“More summer schemes which are active in nature which do not cost lots and cover the whole working day rather than 1 to 2pm/3pm as this again does not suit working parents or means they have to cut their hours and therefore bills become more of an issue.” *(ID 510: female, 41-50y, 1 child, employed full-time)*
	Family activities	There was a desire for inclusive, family-oriented opportunities to support joint participation.	“Any activities that are whole family focused possibly delivered through community centres, civic centres or women’s centres. Particularly low-cost things that include some healthy food/snacks and are on at school holidays and bank holidays would have massive uptake in my opinion. Loads of places are acting as warm spaces and there’s scope for them to act in this capacity too.” (*ID 87: female, 31-40y, 2 children, employed part-time)*
			“Family activities where parents and children can engage in PA together would benefit all. Most activities are aimed at school age children with no provision for toddlers.” (*ID 601: female, 41-50y, 2 children, employed full-time)*
	Classes	Participants highlighted a need for diverse, affordable classes, particularly for older children and non-traditional activities.	“Dance being treated as a form of exercise - especially given it’s known benefits to mental wellbeing and cognitive abilities as well as its contribution to physical fitness.” (*ID 448: female, 41-50y, 1 child, self-employed)*
			“Classes in council community centres for 13+ year olds to encourage exercise, if you don't like camogie/hurling there is nothing to do.” (*ID 564: female, 41-50y, 3 children, employed part-time)*
Gaps and barriers in provision	Rural location and transport	Living in rural areas and limited transport options restricted access to available opportunities	“There isn't anything around me. With no car, a taxi to the local park (as no bus runs that way) costs £19 return. So this would be our event for the month.” (*ID 83: female, 41-50y, 3 children, employed full-time)*
			“We live in the countryside so there are limited opportunities. We have to travel into the main towns to participate in physical activity.” (*ID 708: female, 41-50y, 3 children, employed full-time)*
	Poor maintenance of facilities	Inadequate maintenance and quality of facilities reduced usability and engagement.	“Updated playground, we have to drive to get to a decent one but there's one just down the road, but in poor condition.” (*ID 493: female, 26-30y, 3 children, student)*
			“Public parks being better maintained.” (*ID 216: female, 31-40y, 4 children, employed part-time)*

Abbreviations: GAA: Gaelic Athletic Association; PA: Physical Activity

### Interviews

In total, n = 29 parents/guardians of n = 54 children aged 4–16 years (mean age: 10.3 (SD 4.4) years) participated in the interviews. The demographic characteristics of participants are outlined in Table 1. Two key themes emerged from the analysis of the qualitative interviews 1) What is currently available and who can access it?; and 2) What do parents think needs to change? (see S5 Table and S6 Table for additional information on the main themes and sub-themes).

## Theme 1: What is currently available and who can access it?

### 1.1 Range and types of provision

Aligned with the quantitative data, it was evident that while a range of free/low-cost PA opportunities were available across NI, provision was variable and dependent on the local area and resources. Some parents/guardians perceived their local area to be well-equipped, particularly where strong community club structures existed:

*“The local club structure around us is pretty good for all sports…There’s Gaelic and hockey, soccer, rugby. Our area…[is] well kitted out for free events or low-cost activities. The wee clubs…deliver a good facility to the community.” (ID: 3, Male, 41-50y, 4 children, Employed full-time)*

However, this positive perception was not consistent across all participants. Others described significant gaps in provision, particularly in relation to local authority/council facilities:

“[Names council] *facilities are atrocious. We haven’t even had a swimming pool…The children have missed out on that all summer…All the play parks, you have to pay to get into them, even something as simple as taking them to a forest park for a walk and a play in the park is now costing money.” (ID: 13, Female, 41-50y, 2 children, Employed full-time)*

Some parents/guardians outlined that charges were introduced recently for opportunities that were previously free:

“*A forest park that is within four or five miles of my house that I would have been going to easily 3-4 times a week with my children. In terms of PA for hiking, forest walks, really good playgrounds, open play, open spaces [or] playing with a ball, that has gone from a free activity that we felt was covered under our very expensive [council] rates, now you have to pay £5 in.” (ID: 9, Male, 41-50y, 3 children, Employed full-time)*

In addition to structured provision, informal settings that provide opportunities for unstructured PA, including forest parks, playparks and walkways, were widely recognised as valuable and accessible opportunities for PA. However, concerns were raised regarding the quality, safety and cost associated with some of these environments, which may discourage engagement:

*“Our leisure centre is rubbish, so there's definitely not enough there. Our towns rubbish. Our Council, our Borough is rubbish, so yeah, there's probably not an awful lot in our town…, so we would be sort of limited as to what we could do.” (ID:11, Female, 31-40y, 2 children, Employed part-time)*

Schools were also identified as a key setting for providing equitable access to PA. For some families, schools offered a wide range of opportunities, helping to overcome a lack of provision within the community setting. However, participants acknowledged disparities in provision between schools, suggesting unequal access depending on available school resources, staff and priorities:

“I know we are blessed with an exceptional school that does so much for their PA. They do their Daily Mile, they do everything, they do swimming, they do after school. I see another local school very close that does nothing.”*(ID: 26, Female, 41-50y, 2 children, Employed full-time)*

Findings indicate that while a range of free and low-cost PA opportunities exist, access is uneven and shaped by geographic location, local infrastructure, and school provision, resulting in unequal opportunities for children and adolescents across NI.

### 1.2 Unequal access to existing provision

Despite the availability of free and low-cost PA opportunities, a number of barriers were identified that limited participation. Some parents/guardians remarked positively about the quality of the structured and unstructured opportunities available locally:

*“[We] buy a pass for the local Forest Park here, and just use it plenty and generally just use space where you don't have to pay to go…I suppose it is just kind of saying to them [children] as well ‘where can we go that doesn't cost us money?’” (ID:1, Female, 41-50y, 2 children, Employed part-time)*

However, for other parents/guardians the perceived quality of opportunities and facilities constrained participation even where the opportunities exist:

*“My problem with some of the playgrounds in my area…some of the equipment is out of date, or it's for the wrong ages of children that are in the area. And some of it is just been broken…and that's a problem too, because if your child's favourite equipment is broken, you're not gonna go to that park.” (ID:20, Female, 41-50y, 1 child, Career break).*

Participants also highlighted that free or low-cost provision was not always perceived as advantageous, with some associating higher cost with better quality, potentially discouraging engagement with free/low-cost options:

*“People make the association between cost and quality, and I think that is a challenge. Because we're used to being charged for things now and equating quality to cost and value, I think now being given something free or at a reduced cost, there is issues around that. I think some people may pause or be uncomfortable with that.” (ID:16, Other, 41-50y, 1 child, Unemployed)*

Consistent with this idea, there was perception among a few parents/guardians that children needed to be going to the ‘best’ sports clubs:

*“I think there is a stigma to sending your kids somewhere that is entirely funded or doesn't have the name of an Olympic gold medallist over the door. It's in a leisure centre instead.” (ID:16, Other, 41-50y, 1 child, Unemployed)*

For some families, low-cost opportunities were viewed as an entry point, with children progressing to more competitive or established clubs over time:

*“The wee gymnastics [names child] goes to it's not probably the best, you know out there, but she enjoys it. It's building up those basics and then if she gets real interest in it, you know, then we'll maybe go on and look at something, you know, a bit more serious.” (ID:17, Female, 31-40y, 2 children, Employed part-time)*

Finally, transport and proximity to facilities emerged as an additional barrier. In areas where local provision was lacking, families were required to travel, introducing both practical and financial challenges:

*“I know the council also runs wee tournaments and things but it’s a commute to them so our kids wouldn’t generally engage with them at all. We know they’re on and we know they’re free but we don’t actually get involved, we don’t participate.” (ID: 3, male, 41-50y, 4 children, employed full-time)*

Together, these findings suggest that the availability of free/low-cost opportunities does not necessarily translate into participation, with perceptions of quality, social norms and location influencing engagement in available opportunities.

### 1.3 Suitability of provision

Parents/guardians identified clear differences in how well provision met the needs of children of different ages. In particular, gaps in provision for older children and adolescents were frequently highlighted, with limited opportunities available beyond early adolescence:

*“[For] their age group it’s harder to get soccer camps because the cut off is 13 or 14. So this year's the first year they can't avail of soccer camps in the local area.” (ID: 10, Female, 51-60y, 3 children, Employed full-time)*

This indicates that existing provision may not adequately support continued participation as children move into adolescence. Conversely, limited access to appropriate free or low-cost opportunities for younger children was also reported by some parents/guardians:

*“I think greater access to free or low cost activities for young children. In our area we’re not entitled to Sure Start or any of those sorts of programmes. There’s nothing that we can access that is free or low cost. And obviously private businesses are trying to make a profit, which they should but there’s nothing outdoorsy or like that that we can join for free.” (ID: 2, Female, 31-40y, 2 children, Employed part-time)*

## Theme 2: What do parents think needs to change?

### 2.1 More diverse and appropriate opportunities

Consistent with quantitative findings on perceived access, participants highlighted the need for improvements in facilities and the availability of free/low-cost PA opportunities. There was an emphasis on expanding the range and diversity of provision, with a preference for accessible, varied and non-competitive activities that support engagement. A wide range of activities were suggested, including dance, multisport, gymnastics, running, cycling and walking, with particular emphasis placed on ensuring that activities are enjoyable:

*“I think the fun element needs to be more promoted rather than places always going we need you to come and you need to join us a member and you need to commit. I think maybe more things in the local community that can promote fun and exercise.” (ID: 29, Female, 41-50y, 4 children, Employed full-time)*

Swimming was repeatedly identified as a priority, particularly given that it represents a potentially life-saving skill. Parents/guardians highlighted the importance of ensuring that all children have access to swimming opportunities:

*“I think swimming is very important because that's one that's also like potentially lifesaving. So I do think it's a skill that every child should have…swimming needs to be universal and accessible and viable, and available to everybody and that's not just about cost, but availability. Every kid should be able to go swimming and learn swimming for health and safety.” (ID 16, other, 41-50y, 1 child, Unemployed).*

While organised sport was valued, there was a clear view that future provision should extend beyond structured, competitive activities to include more informal PA opportunities:

*“Not every child likes sport. But there’s bound to be something that every child likes. You know, whether it's out on the bike, playing football, dance, whatever. They probably just need to encourage the child to try something that they do enjoy.” (ID: 22, Female, 31-40y, 3 children, Self-employed)*

This preference for simplicity and accessibility was often reflected in references to traditional, low-cost activities that require minimal equipment and can be delivered in community settings. Several participants provided examples from their own childhood such as tennis, rounders or cricket:

*“I think community based, just like an invite on a Saturday morning and you go. Kids can go here and have a go, practice batting, practice running, practice jumping, just an hour where they can go and just expel [energy].” (ID: 24, Female, 41–50 y, 2 children, Employed part-time)*

Finally, participants highlighted the importance of reducing the financial risk associated with participation in organised sport. The availability of taster and drop-in sessions was viewed as a key mechanism to support initial engagement, allowing children to try activities before parents/guardians make a financial commitment:

*“Last year she did the Triathlon Ireland summer camp. It was 3 days and she loved it, and then she did it again this year and then she did a wee mini triathlon a couple of weeks ago and she loved it. But you know, triathlon is it is an expensive sport. But that sort of taster session…so that you know if your child has a notion to do something and then a month into it is like I don't like this, you haven't paid a whole lot of money or, bought a lot of kit.” (ID: 25, Female, 51–60 y, 3 children, Employed full-time)*

Expanding provision requires not only increasing availability but ensuring opportunities are diverse and aligned with children’s interests, with swimming, informal activities and taster sessions thought to support engagement.

### 2.2 Better local environments and facilities

In addition to expanding the range of activities available, participants highlighted the importance of improving both the accessibility and quality of existing facilities to support family engagement in PA. In particular, council-run facilities were identified as key settings, with parents/guardians emphasising the need to reduce costs and make these spaces more family-friendly:

*“My big thing is the leisure centres. I just think as a family they can offer maybe a bit more.” (ID:19, Female, 41-50y, 4 children, Employed full-time)*

Participants emphasised the importance of local availability, highlighting that increased provision within communities is necessary to ensure equitable access and greater choice for children. A lack of nearby facilities was seen to limit opportunities for participation:

*“I definitely think having access locally to proper facilities would be brilliant. I think having maybe more stuff locally, definitely would be helpful.” (ID: 4, Female, 51–60 y, 1 child, Employed part-time)*

Access to well-maintained, safe and locally available play parks was also identified as essential for supporting regular participation in PA. Participants emphasised the importance of having facilities within walking distance, noting that the absence of suitable local spaces can restrict opportunities for everyday activity:

*“I think to be honest it's free, good open spaces, good facilities that are up to date and fit for purpose. To have decent play parks that are within walking distance. I think it's just access to decent facilities. There's actually no open space like where I live, like there is no actual play park where you could literally just go and like walk your dog or take your kids on a bike.” (ID: 21, Female, 31–40 y, 2 children, Employed part-time)*

However, alongside concerns about availability, safety emerged as a key consideration. Some parents/guardians expressed concerns about allowing children to use community spaces independently, highlighting how perceived safety concerns may limit engagement even where facilities exist:

*“But then there's parents who are fearful. Like where is my wee child going, are they supervised? Are they safe? And then if you start to put that in, you have to start to monitor things. Then you have to pay people if you're going to monitor or there might be insurance. It's just a nightmare and I don't know how you solve these problems.” (ID: 23, Male, 51–60 y, 1 child, Employed full-time)*

These concerns were further linked to broader perceptions of societal change, with some participants suggesting that opportunities for informal, outdoor play have declined over time:

*“I think we're losing that whole social side of things where you used to knock on people's door and go ‘do you want to go out and play?’ You know, because 9 times out 10, the parents are gonna go ‘no, you're not going out to play, there's monsters out there’.” (ID: 15, Female, 41–50 y, 2 children, Employed full-time)*

In response to these challenges, parents/guardians also proposed practical solutions to improve access to safe and local facilities, including the use of existing infrastructure such as schools outside of term time:

*“Local government again, why are we not using our schools? Why are they shut for three months when they have all the all the tools, all the resources…Kids are safe, send the parents up, that is not hard.” (ID: 15, Female, 41–50 y, 2 children, Employed full-time)*

Improving participation in PA requires not only increased provision, but also affordable, accessible and safe environments, with enhanced local facilities supporting regular engagement.

### 2.3 Reducing financial barriers

Participants highlighted the importance of addressing financial barriers to participation to support equitable access to PA. While some parents/guardians acknowledged that certain activities are inherently more expensive and unlikely to be offered free of charge, there was recognition that these costs may limit opportunities for some children:

*“A lot of the stuff that I would like to see, opportunities for kids to do costs so much money, you know like sailing, or equestrian events or water skiing or something like that. You know there would just be no way that it could ever be justified to spend money on something like that as a free initiative.” (ID: 3, Male, 41-50y, 4 children, Employed full-time)*

The majority of participants emphasised the need to increase the availability of low-cost and community-based activities to reduce financial barriers and support wider participation. These types of opportunities were also seen to foster social connections and encourage sustained engagement:

*“I think local community initiatives are really good. If there was things run in the community that were family orientated, like a family fun run or a weekly fun run, things like that that are free and that build up a sense of community and you know that gets friendships going as well. If people make and develop friendships then they're more inclined to go as well.” (ID: 26, Female, 41-50y, 2 children, Employed full-time)*

Beyond cost, parents/guardians highlighted the importance of transparency in pricing and perceived value, suggesting that clearer information about costs may help families feel more confident in their spending decisions:

*“I suppose if parents feel that it's worthwhile like activity, they don't mind paying a bit for it. I think they need to know what those costs are for and what are they paying for and how good of a bargain is it?” (ID: 29, Female, 41-50y, 4 children, employed full-time)*

Participants also identified practical strategies to reduce the financial burden associated with participation in organised sport. These included minimising the need for specialised equipment and improving access through shared resources and transport:

*“I think kind of reducing the need for kids to have kit. Having equipment that can be used by everybody, that you don't have to have even the likes of a hockey stick or you don't have to have shin guards or you know that whoever is delivering [the sport] can provide even just a minimum amount of equipment and the clothing doesn't really matter.” (ID: 28, Female, 41-50y, 2 children, Employed full-time)*

Finally, the provision of discounts or subsidies was viewed as essential to ensure affordability for low-income families, while maintaining a sense of value and commitment. In particular, participants emphasised the importance of targeted support for those living in deprived areas:

*“I think the Council run projects should have…some free spaces for people who really are struggling in deprived areas. I think parents really want their child to take part in activities, especially during the summer, but they just can't afford that extra bit on their budget. And it's sad for people whose children can't take part. And then I think that kind of causes, then a built-in thing in your memory, as I can only play sports if I have money.” (ID: 29, Female, 41-50y, 4 children, Employed full-time)*

### 2.4 Improving awareness and inclusion

Parents/guardians identified several groups for whom current provision of free/low-cost PA opportunities was perceived to be insufficient, highlighting issues of accessibility and inclusivity. In particular, adolescents were frequently described as an underserved group with limited opportunities:

*“There is a loophole for the teenagers like big time. And yeah, you see activities for kids up to 13, 14. You know different wee things during the summer periods. But nothing really afterwards.” (ID: 10, Female, 51-60y, 3 children, Employed full-time)*

Gender-specific barriers were also identified, particularly in relation to adolescent girls. Participants highlighted the need for activities that are more appealing especially for those who may not participate in organised sport:

*“I teach in an all-girls secondary school and most of them are so unfit. It's probably trying to find things that will appeal to them, you know, that sounds so sexist. But is it, I know lots of people aren't good at team sports, I wasn't so do we need more dance type things that are going to appeal the girls?” (ID: 18, Female, 51–60 y, 1 child, Employed part-time)*

In addition to age and gender, parents/guardians emphasised the importance of inclusive provision for children with additional needs. These children were perceived to face unique barriers to participation, particularly in group-based or competitive environments:

*“I work in the world of special needs and children with autism and children who find it difficult to socially interact. So there's a gap for those children that need some kind of, special activity for the children that aren't big in crowds. There's maybe a whole population there that have unmet needs in terms of sport and physical exercises because of the pandemic, they've become socially isolated and socially anxious.” (ID: 7, Female, 41-50y, 1 child, Employed full-time)*

Across participants, accessibility emerged as a key practical consideration. For some families, transport and ease of access to facilities were seen as key barriers, particularly for adolescents who may seek greater independence but whose parents want to ensure they are safe and avoiding trouble:

*“Like can there not be a bus that goes to your leisure centre and it's free and sort of has it route through like a couple of housing estates and picks the kids up and it goes on the hour, every hour in the evening between like, 4:00 PM and 9:00 PM? That would help and I'm sure a lot of parents would be so glad of it because they're worried about what their kids are up to in the evening when they out of the house.” (ID: 18, Female, 51–60 y, 1 child, Employed part-time)*

Finally, a consistent finding across participants was the need for improved awareness and promotion of available opportunities. Even where provision existed, limited visibility was perceived to reduce engagement:

*“I think it's…not knowing and not being involved in the likes of the Leisure Centre to get the flyers. We follow them on Facebook, but then I don't know who's responsibility ability that is [to promote events/activities]. There's definitely loads of things out there. It is maybe just better organisation, you know, advertising. Maybe more of that that support to get parents along to it.” (ID: 17, Female, 31-40 y, 2 children, Employed part-time)*

Additional information relating to this theme can be found in S6 Table.

The Health Equity Framework [18] was used as a conceptual lens to interpret the findings. Factors identified during this study are mapped onto the four interacting spheres of the framework. Cross-cutting factors may operate across multiple spheres (Fig 1).

**Fig 1:**
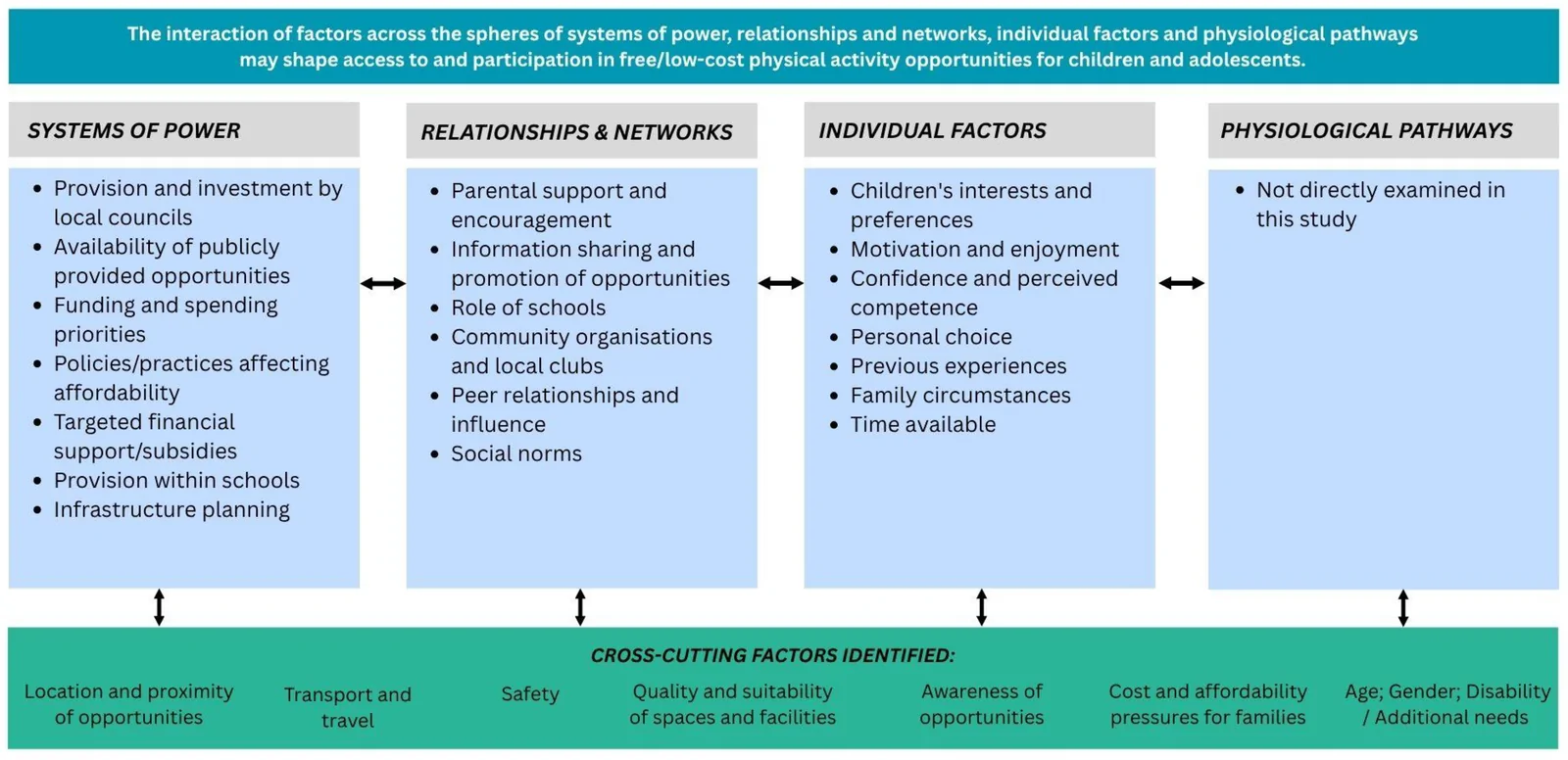
Health Equity Framework: conceptual interpretation of factors influencing access to free/low-cost physical activity opportunities. Adapted from Peterson et al. (2021).

## Discussion

### Children’s engagement with free/low-cost PA opportunities

The aim of this study was to explore parental/guardian perspectives on children’s engagement with free/low-cost PA opportunities, factors affecting participation, the availability of such opportunities locally, and views on future provision. Among this cohort, there was high engagement in free/low-cost PA opportunities, with walking, running and outdoor play being the most commonly reported activities. This likely reflects the accessibility and low financial burden associated with these activities, particularly within the context of the cost-of-living crisis [10]. These findings are consistent with previous research which reports that informal and unstructured activities (for example active play, walking and cycling) represent key contributors to children’s overall PA levels [32].

### Factors affecting participation

The findings can be understood through the Health Equity Framework [18], which emphasises the importance of fair access to resources and opportunities for health and recognises that health is influenced by interacting factors across multiple levels. While the majority of parents/guardians reported that their children regularly participate in free/low-cost PA activities, there was some variation in engagement with these opportunities. Higher participation in free/low-cost PA opportunities was associated with increased parental/guardian support for PA which draws similarities to existing research on the importance of the home environment and family support in promoting PA for children and adolescents [33]. Interestingly, within the present study there were no differences in participation in free/low-cost PA opportunities by most indicators of SES, including household income and deprivation tertile. Although there were differences based on level of education (P = 0.024), the results did not show a clear socioeconomic gradient. These results were somewhat unexpected given that previous research reports higher PA participation and a greater likelihood of meeting PA guidelines among individuals of higher SES [12]. One possible explanation for this is that the ongoing cost-of-living crisis may have encouraged families across all socioeconomic backgrounds to rely more heavily on free/low-cost opportunities as substitutes for structured, paid activities [34], rather than an absence of underlying inequalities. Consistent with this, it has been previously reported that among parents/guardians in NI, 29% of parents/guardians said their children have started/increased the number of free activities that they do (such as walking and running) [5,35]. While free/low-cost opportunities may reduce financial barriers to PA, our findings suggest that financial cost is only one aspect of access with location, transport, availability and quality of opportunities also influencing participation. It is this interaction of individual, social and environmental factors that appears to influence whether opportunities are accessible and suitable for families.

Despite high levels of participation in free/low-cost PA opportunities, the majority of parents/guardians in NI (76%) perceived there to be insufficient provision for children and adolescents in their local area. This discrepancy between high levels of participation and perceptions of insufficient provision highlights that participation in some free/low-cost activities does not necessarily indicate equitable access to a sufficient range of opportunities. Within this research, gaps in provision were evident for particular groups including adolescents, girls and children with additional needs. These findings align with existing literature demonstrating that girls engage in less PA than boys and are less likely to meet recommended guidelines [36,37] Furthermore, PA levels decline with age, with evidence showing substantial reductions during adolescence, particularly among girls [38–42]. Similarly, children and adolescents with disabilities or additional needs are consistently reported to be less physically active than their peers and face additional barriers including limited access to appropriate facilities, higher costs, transport challenges, and a lack of tailored provision [43,44]. Addressing these disparities requires tailored, inclusive approaches that meet the needs of all children and adolescents.

### Local provision

Qualitative findings highlighted perceived inequalities in local provision, particularly in relation to the accessibility, affordability and quality of facilities across different areas. This reflects wider evidence suggesting that individuals living in more deprived areas are more likely to live in neighbourhoods with less access to green space [45]. In NI, it has previously been estimated that one in five children living in severe poverty did not have access to local, safe play areas [46]. More recent data from Playboard NI found that one in three children in NI lacked sufficient places to play [35]. While no differences in participation in free/low-cost PA opportunities based on index of multiple deprivation were observed within the current study, there is a need to profile and better understand the availability of such opportunities across communities in NI, particularly as evidence from other UK regions shows that the availability of PA facilities generally declines as levels of deprivation increase [47].

Council-run facilities were identified as key settings, with parents/guardians emphasising the need to reduce costs and make these spaces more family-friendly. Previous research suggests that partnerships with local authorities (councils) to provide children with free swimming access can increase participation and may help reduce inequalities in PA [48]. Evidence suggests policy change is more feasible at the local level, particularly when councils have the capacity to act on evidence [49].

Beyond affordability, participants emphasised the importance of local availability, noting that limited proximity to facilities can restrict opportunities for participation. In addition to community settings, schools play a critical role in providing free/low-cost PA opportunities for children and adolescents [50]. This is supported by a substantial evidence base highlighting the importance of school-based PA for educational and health outcomes [51–54]. However, participants in this study highlighted variation in school-based provision, suggesting that access remains inconsistent. Within this study, participants identified school buildings and infrastructure as a potential setting for PA outside of term time. This warrants further investigation. Together, these results and existing evidence underline the importance of further investigating PA provision across a range of settings in NI to ensure there is equitable access across the region [7].

### Future provision

While the majority of parents/guardians said their children participated in free/low-cost activities, they perceived the marketing and promotion of these opportunities to be insufficient, resulting in low awareness of what is available within local communities. Efforts to promote PA among families should therefore consider improving the visibility of opportunities, particularly as parental support is positively associated with children’s PA levels and parents are often described as key ‘gatekeepers’ of children’s PA [55]. These findings align with previous qualitative research demonstrating that limited awareness of opportunities can act as a barrier to engagement [56]. Looking through the lens of the Health Equity Framework [18], in particular the systems of power, it would appear the issue isn’t simply that parents fail to find activities. Rather, the way opportunities are organised, funded, promoted and distributed influences whether families can access them. From a health equity perspective, improving awareness represents an important but insufficient intervention if looked at in isolation. A centralised platform may support parents’ role as gatekeepers to children’s PA, but equitable participation also requires provision that is affordable, flexible, locally available and responsive to children's interests. Sustained investment, infrastructure and policy support are therefore necessary to address the structural conditions that shape families’ opportunities to engage in PA [56,57].

In addition, while parents/guardians were cognisant of the challenges this would bring, they suggested targeted supports for low-income families, such as means-tested subsidies as low-income families are frequently described as a ‘hard to reach’ population [58]. Children from these households are less likely to engage in regular PA [6,11,12], and may face additional barriers to participation, included limited access to suitable and affordable opportunities. These findings highlight the importance of equitable and inclusive provision, rather than assuming that universally available free provision will produce equitable participation.

### Strengths and limitations

To the authors’ knowledge, this is the first study to use a mixed-methods design to explore engagement in and perceptions of free/low-cost PA opportunities for children and adolescents. The findings of this study will be of relevance to those involved in service provision, resource/budget allocation, local councils/authorities and policymakers. Despite, the majority of children participating in free/low-cost PA opportunities, there is a paucity of research on free/low-cost provision and the potential for free/low-cost opportunities to address inequality gaps in PA. The mixed-methods design was a strength of this study and allowed for a deeper exploration and more nuanced analysis. Although the study was conducted in NI specifically, the findings will be generalisable to other UK regions and countries impacted by the cost-of-living crisis. While participants were purposively sampled across deprivation tertiles to ensure socioeconomic diversity, it must be acknowledged that for both the survey and the interviews the majority of participants were female and in employment, with the majority having a higher level of education. In addition, as a non-probability, social media and snowball sampling approach was used, the sample may not be representative of the wider population and is subject to potential selection bias [58,59]. Further investigation of cost as a barrier to participation in PA among low-income families is therefore required as the findings of this study are likely to reflect a ‘better-case-scenario’.

The inclusion criteria for this study encompassed parents/guardians of children aged 4–16 years, resulting in a wide age range. This may represent a limitation, as PA preferences, and opportunities differ substantially between younger children and older adolescents. However, existing evidence suggests that participation in organised sport begins in early childhood and varies by socioeconomic status [60,61]. Including parents of younger children therefore enabled exploration of participation in free/low-cost PA opportunities across developmental stages. Future research may benefit from stratifying findings by age group to better understand age-specific needs and patterns of participation.

It is possible that the questions asked within the survey and interviews which used the term ‘cost-of-living crisis’ may have biased participant responses as parents/guardians were detailing their experiences of an already defined ‘crisis’. In addition, frequency of engagement in free/low-cost PA opportunities was self-reported by parents/guardians and may be subject to measurement or recall bias. Future research should consider the inclusion of device-measured PA and objective environmental data, such as Geographic Information Systems (GIS) based assessment of local facilities and opportunities. Finally, as with all interview-based research, responses may have been influenced by recall error or social desirability bias. However, the integration of quantitative and qualitative findings allowed for a more comprehensive understanding of both patterns of participation and the contextual factors shaping access and engagement.

## Conclusion

Despite high levels of engagement in free/low-cost PA opportunities, this study highlights important gaps in provision, accessibility and awareness. While informal activities such as walking and outdoor play were popular, most parents perceived there to be insufficient provision for children and adolescents within their local area. Structural issues including limited local availability, poor facilities and low awareness were identified as barriers to participation, particularly for adolescents, girls and children with additional needs.

Findings from this mixed-methods study suggest that improving participation in free/low-cost PA opportunities is a complex issue which requires more than increasing provision alone. Free/low-cost provision may reduce financial barriers to participation, but our findings suggest that access is also influenced by factors including local availability, transport, quality, suitability and awareness of opportunities. These findings highlight the importance of considering the multiple and interacting factors that influence access to and participation in free/low-cost PA opportunities. Therefore, a coordinated approach is needed which encompasses a more extensive range of provision, improved facilities, enhanced affordability and targeted financial support for families experiencing socioeconomic disadvantage.

Free/low-cost PA opportunities represent a valuable and potentially scalable strategy to support children’s PA, particularly within the context of the ongoing cost-of-living crisis. However, the potential to support equitable PA participation depends not only on whether opportunities are free or affordable, but also on whether they are accessible, suitable and known to families.

## Supporting information

S1 Table Interview topic guide on free/low-cost PA opportunities. (DOCX)

S2 Table Differences in participation in free/low-cost physical activity opportunities by measures of socioeconomic status. (DOCX)

S3 Table Differences in perceived access to free/low-cost physical activity opportunities by parental support for physical activity and access to sports equipment at home. (DOCX)

S4 Table Perceived access to free/low-cost physical activity opportunities across measures of socioeconomic status. (DOCX)

S5 Table Illustrative interview quotations supporting Theme 1: Current provision of free and low-cost physical activity opportunities. (DOCX)

S6 Table Illustrative interview quotations supporting Theme 2: Improving provision and access to free and low-cost physical activity opportunities. (DOCX)
