# Supplementary material for: Free/low-cost physical activity opportunities for children and adolescents: Parental perspectives on participation, access and provision
Source: PLOS Glob Public Health. 2026 Nov 3;6(11):e0005770. doi: 10.1371/journal.pgph.0005770 (PMC13730368; doi:10.1371/journal.pgph.0005770)
Supplement: Supplementary file 1 [file pgph.0005770.s007.pdf]

Review Comments:

This study addresses an important and timely topic and takes on a challenging methodological task by seeking to understand children's experiences through parental perspectives. The authors provide a thoughtful contribution by exploring this perspective and by combining several methodological approaches. However, the manuscript would benefit from further clarification and refinement, particularly regarding the role and treatment of the questionnaire data.

Introduction:

Overall, the introduction is rather long and would benefit from being substantially shortened to achieve a clearer and more focused narrative. Several themes and topics are repeated across sections and could be consolidated into a single, more coherent paragraph. This would strengthen the conceptual clarity of the introduction and improve readability without losing essential context.

Specific comments to the introduction:

Line 33-34: For clarity, I recommend explicitly stating the age group for primary and post primary pupils, and work improving the readability of the sentence.

Line 41-42: The authors state that this phenomenon is well established in the literature; however, this claim is currently insufficiently substantiated. If the phenomenon is indeed well documented, the argument should be supported by additional and more comprehensive references.

Line 53-55: It is unclear whether a change of 3% should be considered substantial in this context. The subsequent sentences appear to provide a stronger and more convincing justification for the authors' argument.

Line 59-67: This section would benefit from substantial rephrasing, as it is currently difficult to follow and appears conceptually cluttered. Clarifying the key message, improving the logical flow, and streamlining the argumentation would greatly enhance readability and strengthen the overall contribution of the paragraph.

Line xxxx: forholder e sig engentlig til ulighedsperspektivet

Materials and Methods:

The quantitative component of the study appears insufficiently developed and somewhat uncertain in its current form. Methodological transparency is limited, particularly with regard to sample size and the handling of small subgroups. Reporting results as “n < 5” raises concerns about both statistical robustness and interpretability, as it complicates the assessment of the precision and reliability of estimates. A clearer justification of the quantitative approach, including sampling procedures, data handling, and analytic strategy, would strengthen the methodological rigor of the paper.

Line 104-105: I would encourage the authors to provide a more detailed description of their inclusion criteria. At present, it remains unclear who was actually eligible to participate in the study, and whether, for example, older siblings could in principle also have responded on behalf of the target child. Clarifying these criteria (including who was invited, who was allowed to respond, and how potential ambiguities within families were handled) would considerably strengthen the transparency and interpretability of the findings.

Line 105: Have you considered allowing the older children to complete the questionnaire themselves rather than relying solely on proxy reports from parents?

Line 111-118: as a reader one is left with the feeling that important information is missing. I would therefore encourage the authors to further develop and clarify this section.

Line 121: Elaborate on which variables you are referring to here.

Line 127-130: Consider moving these lines up to the section where you describe the parents.

Line 130-134: Are these the SES variables you refer to earlier in the manuscript? If so, please harmonize the two sections.

Line 141: Please elaborate on why you have grouped "never" together with "once or twice a month" into the same category. There is a substantial difference between these response options, and I suggest considering three separate categories with " once or twice a year and never" as its own distinct group to improve clarity and analytical precision.

Line 164-167: You fail to report whether this was actually achieved

Line 171, line 185: Be consistent in whether you write "MS Team" or "Microsoft Teams"

Line 170: Remove the following text; PhD), a female academic with previous experience in qualitative research. It is not necessary.

Line 175: Please elaborate on whether the same probe questions were used across all interviews or if new ones were developed each time.

Line 193-195: Could you please clarify what specific transformations were applied to the variables that violated normality assumptions, and provide more detail on how you determined that the transformed data still remained skewed in some cases? The current description is ambiguous and makes it difficult to assess the appropriateness of switching to non-parametric tests. For example, which variables were affected, what transformation methods (e.g., log, square root) were used, and what criteria (e.g., Shapiro-Wilk test post-transformation) led to the final choice of analytic approach?

Line 200-202: Please elaborate on what additional information is being collected here?

Results:

Overall, the section on questionnaire results requires substantial revision for clarity, structure, and completeness. I recommend thoroughly rewriting it, drawing inspiration from how similar studies have presented their findings—both in terms of writing style and the specific details reported (e.g., response rates, descriptive statistics by subgroup, effect sizes, and clear links to research questions). Additionally, there is a missing "=" between "n" and the number in several places throughout the text.

Line 224: How many individuals were invited to participate in the questionnaire, and do you have a response rate? This would help indicate whether the 885 participants are representative or not.

Line 229-232: Avoid using "majority" in two consecutive sentences; please rephrase for improved variety in wording.

Line 236 (Table 1): The table appears to describe the participation process, but this is unclear without further context from the main text. Tables should be fully self-explanatory and interpretable independently of the manuscript body, with comprehensive footnotes, clear headings, and defined abbreviations to ensure standalone readability. Additionally, quantitative data for subgroups with fewer than 5 individuals should not be reported, as it compromises data anonymity, makes the dataset overly identifiable, and undermines the strength and robustness of the data.

Line 251-253: Rephrase to make it easier to understand.

Line 256-259: The parenthetical statements are confusing for readers and obscure the intended meaning. Please rephrase to better assist readers in understanding the connection and flow without requiring additional effort to parse.

Line 270-276: You are missing your own data/numbers—what do they show? This is the information you should present here.

Interviews:

Consider removing the overview section or incorporating direct quotes into it to substantiate the claims, then deleting the subsequent section. Generally, the current approach is thin, as each claim is supported by only a single quote despite having 29 respondents

Line 284: Did you achieve the target of 10 participants from each tertile? If not, please clarify. If so, did they represent 10 from each group? I could not find this information in Table 2.

Line 285: MS Teams (n=21; 72%) and the mean interview duration was 29 (SD =9)

Discussion

The discussion largely repeats the results without engaging substantively with the existing literature. It should instead address whether your findings align with or contradict prior research, highlighting consistencies, discrepancies, and their implications for the field.

Further the manuscript does not currently address the very wide age range of the children about whom parents are reporting. As a reader, it is impossible to discern whether the majority of the sample consists of younger or older children, which may substantially influence how parents perceive and respond to questions about needs and use of free services. It seems likely that the needs and relevant offers for a 4-year-old differ markedly from those of a 16-year-old, and this heterogeneity may have important implications for the interpretation of the findings. I would therefore encourage the authors to describe and reflect more explicitly on the age distribution of the child sample and to consider whether, and how, age might shape parents' responses and the overall conclusions of the study

Line 473-474: Can your data support making claims about this?

Line 485-487: Here, you contradict yourselves, as you have just stated earlier that there was no difference according to your data.

Line 488-496: All of this is a repetition of your results section. This should be rewritten to instead discuss what the literature says about this topic in relation to your findings—does it support them, or is there disagreement?

Line 499-500: A reference is missing here.

Line 506-520: All of this is a repetition of your results section. This should be rewritten to instead discuss what the literature says about this topic in relation to your findings—does it support them, or is there disagreement?

Line 548-549: Be cautious not to draw conclusions that your own data cannot support.

Line 551: I would encourage the authors to use a different term instead of "whole-system approach," as it has not been mentioned previously in the manuscript and therefore appears confusing when suddenly introduced in the conclusion.
