# Supplementary material for: Free/low-cost physical activity opportunities for children and adolescents: Parental perspectives on participation, access and provision
Source: PLOS Glob Public Health. 2026 Nov 3;6(11):e0005770. doi: 10.1371/journal.pgph.0005770 (PMC13730368; doi:10.1371/journal.pgph.0005770)
Supplement: Supplementary file 2 [file pgph.0005770.s001.docx]

**S1 Table:** Interview topic guide on free/low-cost PA opportunities.

| 1. | In NI, only 1 in 5 children do enough physical activity. As a parent/guardian, have you any thoughts on why this might be? |
| --- | --- |
| 2. | What would help your family be more active? |
| 3. | Does the cost of sport or physical activity act as a barrier for your child/children? Do you think there is a need for more free or low-cost activities for children and young people? |
| 4. | Are you aware of any free or low-cost physical activity opportunities in your local area? Do you or your children use any of these facilities? |
| 5. | What types of free or low-cost physical activity opportunities would you like to see in your community? |
