# Supplementary material for: Free/low-cost physical activity opportunities for children and adolescents: Parental perspectives on participation, access and provision
Source: PLOS Glob Public Health. 2026 Nov 3;6(11):e0005770. doi: 10.1371/journal.pgph.0005770 (PMC13730368; doi:10.1371/journal.pgph.0005770)
Supplement: Supplementary file 3 [file pgph.0005770.s002.docx]

**S2 Table:** Differences in participation in free/low cost physical activity opportunities by measures of socioeconomic status

|  | Participation in low or no cost physical activity opportunities | | | | | | P* |
| --- | --- | --- | --- | --- | --- | --- | --- |
|  | Below average | | Average | | Above average | |  |
|  | n | % | n | % | n | % |  |
| **Household income** |  |  |  |  |  |  |  |
| Low (<£29,999 pa) | 68 | 36.8 | 41 | 22.2 | 76 | 41.1 | 0.421 |
| High (>£30,000 pa) | 261 | 42.3 | 113 | 18.3 | 243 | 39.4 |  |
| Prefer not to say | 20 | 37.7 | 14 | 26.4 | 19 | 35.8 |  |
| **Index of multiple deprivation ^§^** |  |  |  |  |  |  |  |
| Most deprived | 87 | 39.7 | 41 | 18.7 | 91 | 41.6 | 0.953 |
| Average | 147 | 41.1 | 72 | 20.1 | 139 | 38.8 |  |
| Least deprived | 103 | 41.7 | 50 | 20.2 | 94 | 38.1 |  |
| **Education Level** |  |  |  |  |  |  |  |
| Primary / secondary school up to 16 years | 30 | 36.6 | 16 | 19.5 | 36 | 43.9 | 0.024 |
| Further education | 49 | 30.2 | 39 | 24.1 | 74 | 45.7 |  |
| Higher education | 270 | 44.2 | 113 | 18.5 | 228 | 37.3 |  |
| **Locality** |  |  |  |  |  |  |  |
| Urban | 139 | 42.0 | 79 | 22.1 | 119 | 36.0 | 0.485 |
| Rural | 168 | 39.7 | 78 | 18.4 | 177 | 41.8 |  |
| Mixed urban/rural | 30 | 42.9 | 12 | 17.1 | 28 | 40.0 |  |
| **Family structure** |  |  |  |  |  |  |  |
| Care of children shared with partner, ex-partner or grandparent | 299 | 40.5 | 138 | 18.7 | 301 | 40.8 | 0.093 |
| Care of children - single parent | 50 | 42.7 | 30 | 25.6 | 37 | 31.6 |  |
| **Benefit entitlement** |  |  |  |  |  |  |  |
| Someone within the household receives benefits | 105 | 42.7 | 54 | 22.0 | 87 | 35.4 | 0.107 |
| No-one within the household receives benefits | 243 | 40.1 | 112 | 18.5 | 251 | 41.4 |  |
| **Employment status** |  |  |  |  |  |  |  |
| Unemployed / stay at home parent / student / carer / retired | 30 | 35.7 | 15 | 17.9 | 39 | 46.4 | 0.249 |
| Employed / self-employed | 299 | 41.7 | 137 | 19.1 | 281 | 39.2 |  |
| Multiple job holding | 20 | 37.0 | 16 | 29.6 | 18 | 33.3 |  |

*Differences between groups were assessed using Chi-Square test

§ Tertile 1 – Most deprived; 0-296; Tertile 2 – Average; 297-593; Least deprived; 594-890

Abbreviation: pa; per annum
