# Supplementary material for: Free/low-cost physical activity opportunities for children and adolescents: Parental perspectives on participation, access and provision
Source: PLOS Glob Public Health. 2026 Nov 3;6(11):e0005770. doi: 10.1371/journal.pgph.0005770 (PMC13730368; doi:10.1371/journal.pgph.0005770)
Supplement: Supplementary file 4 [file pgph.0005770.s003.docx]

**S3 Table:** Differences in perceived access to free/low cost physical activity opportunities by parental support for physical activity and access to sports equipment at home

|  | Perceived access to low/no cost physical activity opportunities | | | |  |
| --- | --- | --- | --- | --- | --- |
|  | Enough opportunities | | Not enough opportunities | | P* |
|  | Median | 25th-75th percentile | Median | 25th-75th percentile | |
| Logistical support | 11.5 | 9.0-12.0 | 11.0 | 9.0-12.0 | 0.094 |
| Modelling | 9.0 | 7.8-11.0 | 9.0 | 7.0-11.0 | 0.424 |
| Use of community resources | 10.0 | 8.0-11.3 | 9.0 | 8.0-11.0 | 0.124 |
| Limiting sedentary behaviour | 9.0 | 8.0-11.3 | 9.0 | 9.0-11.0 | 0.576 |
| Total parental support for PA | 39.0 | 34.0-43.0 | 38.0 | 33.8-42.0 | 0.206 |
| Access to sports equipment at home | 9.0 | 7.0-11.0 | 8.0 | 6.0-10.0 | 0.011 |

*Differences between groups assessed using the Mann-Whitney U Test
