# Supplementary material for: Free/low-cost physical activity opportunities for children and adolescents: Parental perspectives on participation, access and provision
Source: PLOS Glob Public Health. 2026 Nov 3;6(11):e0005770. doi: 10.1371/journal.pgph.0005770 (PMC13730368; doi:10.1371/journal.pgph.0005770)
Supplement: Supplementary file 5 [file pgph.0005770.s004.docx]

**S4 Table:** Perceived access to free/low cost physical activity opportunities across measures of socioeconomic status

|  | Perceived access to low/no cost physical activity opportunities | | | | | |  |
| --- | --- | --- | --- | --- | --- | --- | --- |
|  | Enough opportunities | | Not enough opportunities | | Don't know | | P* |
|  | n | % | n | % | n | % |  |
| **Household income** |  |  |  |  |  |  |  |
| Low (<£29,999 pa) | 12 | 6.5 | 151 | 81.6 | 22 | 11.9 | 0.132 |
| High (>£30,000 pa) | 71 | 11.5 | 457 | 74.1 | 89 | 14.4 |  |
| Prefer not to say | 5 | 9.4 | 44 | 83.0 | 4 | 7.5 |  |
| **Index of multiple deprivation^† §^** |  |  |  |  |  |  |  |
| Most deprived | 24 | 11.0 | 175 | 79.9 | 20 | 9.1 | 0.054 |
| Average | 34 | 9.5 | 275 | 76.8 | 49 | 13.7 |  |
| Least deprived | 29 | 11.7 | 137 | 70.0 | 45 | 18.2 |  |
| **Education Level** |  |  |  |  |  |  |  |
| Primary / secondary school up to 16 years | 2 | 2.4 | 69 | 84.1 | 11 | 13.4 | 0.051 |
| Further education | 12 | 7.4 | 129 | 79.6 | 21 | 13.0 |  |
| Higher education | 74 | 12.1 | 454 | 74.3 | 83 | 13.6 |  |
| **Locality** |  |  |  |  |  |  |  |
| Urban | 33 | 10.0 | 252 | 76.1 | 46 | 13.9 | 0.849 |
| Rural | 45 | 10.6 | 317 | 74.9 | 61 | 14.4 |  |
| Mixed urban/rural | 9 | 12.9 | 54 | 77.1 | 7 | 10.0 |  |
| **Family structure** |  |  |  |  |  |  |  |
| Care of children shared with partner, ex-partner or grandparent | 82 | 11.1 | 556 | 75.3 | 100 | 13.6 | 0.126 |
| Care of children - single parent | 6 | 5.1 | 96 | 82.1 | 15 | 12.8 |  |
| **Benefit entitlement** |  |  |  |  |  |  |  |
| Someone within the household receives benefits | 13 | 5.3 | 210 | 85.4 | 23 | 9.3 | <0.001 |
| No-one within the household receives benefits | 75 | 12.4 | 439 | 72.4 | 92 | 15.2 |  |
| **Employment status** |  |  |  |  |  |  |  |
| Unemployed / stay at home parent / student / carer / retired | 3 | 3.6 | 74 | 88.1 | 7 | 8.3 | 0.085 |
| Employed / self-employed | 78 | 10.9 | 537 | 74.9 | 102 | 14.2 |  |
| Multiple job holding | 7 | 13.0 | 41 | 75.9 | 6 | 11.1 |  |

*Differences between groups were assessed using Chi-Square test

§ Tertile 1 – Most deprived; 0-296; Tertile 2 – Average; 297-593; Least deprived; 594-890

Abbreviation: pa; per annum
