# Supplementary material for: Free/low-cost physical activity opportunities for children and adolescents: Parental perspectives on participation, access and provision
Source: PLOS Glob Public Health. 2026 Nov 3;6(11):e0005770. doi: 10.1371/journal.pgph.0005770 (PMC13730368; doi:10.1371/journal.pgph.0005770)
Supplement: Supplementary file 6 [file pgph.0005770.s005.docx]

**S5 Table:** Illustrative interview quotations supporting Theme 1: What is currently available and who can access it?

| **Sub-theme** | **Description** | **Illustrative Quote** |
| --- | --- | --- |
| Range and types of provision | Council-led initiatives can provide affordable opportunities, but access and quality vary across areas. | “Swimming in your local leisure centre, for the summer is down to a £1 for a child. You know, that's fantastic. So, we have been trying to use the most of it and then the two-year-old gets in for free.”  *(ID: 17, Female, 31-40 y, 2 children, Employed part-time)* |
|  | Availability of local facilities is inconsistent, with some areas experiencing significant gaps in provision. | "Newry & Mourne District Council's facilities are atrocious. We haven't even got a swimming pool. The swimming pool, which was supposed to be state-of-the-art, closed in March and hasn't re-opened. So we've gone now a whole summer for what would have been a very feasible activity for parents, you know it's cheap but it isn't there. So the children have missed out on that all summer."  *(ID: 13, Female, 31-40 y, 2 children, Employed full-time)* |
|  | Schools act as key providers of accessible and often free physical activity opportunities for children. | "The school is very good at offering activities, not that we pay for a lot of it. [There's] after-school things but they also offer a lot of free stuff within the school, so we’re very lucky"  *(ID: 22, Female, 31-40y, 3 children, Self-employed)* |
| Unequal access to existing provision | Limited awareness of available opportunities reduces engagement, even where there is provision. | “There's definitely loads of things out there...Maybe more of that support to get parents along to it. Free activity wise, I'm sure if I looked, I could probably find it. I think it really does come back to just being aware of it.”  *(ID: 17, Female, 31-40 y, 2 children, Employed part-time)* |
|  | A lack of suitable free/low-cost opportunities creates financial barriers to sustained participation. | "The cost is increasing like we are going to have to spend money definitely to keep her active. Because there's nothing here. Her camogie and football is all finished until next February, March, so we're going to have to financially pay for her to exercise. Like yes, we can go for a walk as a family and we do that, but it probably wouldn't be enough for her like she's an active child and would want to remain active. But there's no local free gym class. There's no gymnastics, there's nothing for her age group."  *(ID: 26, Female, 41-50 y, 2 children, Employed full-time)* |
|  | Inadequate facilities impacts the quality and availability of opportunities. | "It's the lack of green areas for kids to partake in activities. Like I know some very affluent clubs in Belfast ehh Gaelic clubs and they just don't have the land, they have the numbers, but you know you're relying on a one hour slot once a week. Trying to train under 16 and under 18s. It's just not enough."  *(ID: 23, Male, 51-60 y, 1 child, Employed full-time)* |
|  | Perceived quality influences engagement with available activities. | "I think there has creeped in an element of, not just am I providing my kid with good, healthy activities? But am I, providing them with good enough versions of that said activity. I see that quite a lot, what gym are you at? Not just they're doing gymnastics, but where you going, you know?"  *(ID: 16, Other, 41-50 y, 1 child, Unemployed)* |
|  | Transport costs act as a barrier to participation where local provision is limited. | "I definitely think having access locally to proper facilities would be brilliant do you know. We live in a part of Derry, which is expanding, it’s getting bigger, bigger and bigger. Theres more houses, but there’s no amenities here locally you know. So you have to get into the car to travel, even though it’s only a couple of miles, you know or you get the bus."  *(ID: 4, Female, 51-60 y, 1 child, Employed part-time)* |
| Suitability of provision | An appreciation of the opportunities available can shape engagement and participation. | "Our kids are quite switched on and we have reasonably good access to local walks and parks and nature reserves. One of the big things for kids is they need to learn to appreciate that [however] a lot of kids don’t appreciate that. If there’s an appreciation of that, they can actually realise there’s loads of free stuff to do, without having to spend money on it.”  *(ID: 3, Male, 41-50 y, 4 children, Employed full-time)* |
|  | Provision gaps exist for older children and teenagers, limiting age-appropriate opportunities. | “I just find there's a loop in a lot of activities for teenagers that are over 13 years plus. I've even seen in soccer; my sister sees it at Gaelic as well for that age bracket. Even teams, particularly over sixteens to say 20-year-olds, there's no teams for them children.”  *(ID: 10, Female, 51-60y, 3 children, Employed full-time)* |
|  | Limited access to free or low-cost opportunities restricts participation for younger children in some areas. | "I think greater access to free or low cost activities for young children. In our area we’re not entitled to Sure Start or any of those sorts of programmes. There’s nothing that we can access that is free or low cost. And obviously private businesses are trying to make a profit, which they should but there’s nothing outdoorsy or like that that we can join for free."  *(ID: 2, Female, 31-40 y, 2 children, Employed part-time)* |
