# Supplementary material for: Free/low-cost physical activity opportunities for children and adolescents: Parental perspectives on participation, access and provision
Source: PLOS Glob Public Health. 2026 Nov 3;6(11):e0005770. doi: 10.1371/journal.pgph.0005770 (PMC13730368; doi:10.1371/journal.pgph.0005770)
Supplement: Supplementary file 7 [file pgph.0005770.s006.docx]

**S6 Table:** Illustrative interview quotations supporting Theme 2: What do parents think needs to change?

| **Sub-theme** | **Description** | **Quote** |
| --- | --- | --- |
| More diverse and appropriate opportunities | Increased provision of affordable and accessible swimming opportunities is viewed as a priority, particularly given its importance as a life skill. | "Swimming, I would say would be a big one. I think it's something that all children should know how to do because we do live on an island, you're gonna be near water one way or another. And I don't want a child out there drowning, because they don't know how to swim. So I think that would be a very key one that we would need."  *(ID: 20, Female, 41-50 y, 1 child, Career break)* |
|  | Future provision should extend beyond organised sport to include accessible, informal and everyday PA opportunities. | "I think access to parks, access to green space, access to you know areas to play. Physical activities just doesn't mean like you're playing a sport. It could be you're going for a walk."  *(ID: 8, Female, 41-50 y, 1 child, Employed full-time)* |
|  | The availability of free taster and drop-in sessions may support initial engagement by reducing financial risk and barriers associated with commitment. | "There's a lot of the activities that we've looked into, they don't even offer a taster course to even see if he likes it. You have to sign up for like a month's time. And if he literally goes to one class and hates it, there's £50, £70 whatever it might be that I know I don't have, and he doesn't like what it is that I've taken him to, and I can't force him to go to something that he doesn't want to do because then that's gonna be detrimental later too."  *(ID: 20, Female, 41-50 y, 1 child, Career break)* |
| Better local environments and facilities | Improving the affordability and accessibility of council facilities is viewed as important to support family engagement in PA. | "I think it's the likes of, reducing the cost of getting into the swimming pools so that a family can go for less than £10 as a treat on a Saturday, but that just isn't the case at the minute. So it's just making things more family friendly and more accessible."  *(ID: 13, Female, 31-40 y, 2 children, Employed full-time)* |
|  | Increased local PA provision and improved facilities across communities are needed to ensure equitable access and greater choice for children | "I definitely think more clubs and activities that are available to all communities. We live in South Belfast so people think, oh well, we don't need any clubs here because you know South Belfast has a sort of middle class thing, but I don't agree with that. I think we need them just as much you know or more. We definitely need more choice for kids to be involved in things. I think that would make a big difference, if something were to come up in the future."  *(ID: 19, Female, 41-50 y, 4 children, Employed full-time)* |
|  | Access to well-maintained, safe and locally available play parks is considered essential to support regular engagement in PA. | "I think to be honest it's free, good open spaces, good facilities that are up to date and fit for purpose. To have decent play parks that are within walking distance. I think it's just access to decent facilities. There's actually no open space like where I live, like there is no actual play park where you could literally just go and like walk your dog or take your kids on a bike or you know and then have to say go to a play park all in the same place. I think it's access to parks that's definitely needed."  *(ID: 21, Female, 31-40 y, 2 children, Employed part-time)* |
|  | Greater availability of accessible green and outdoor spaces is needed to provide opportunities for informal and unstructured PA. | "I think in many ways an access to amenities is what I would say. You know, access to parks, access to green space, access to areas to play. Aye access to space, you know, like outdoorsy places. There's no real outdoorsy places where a child can just run and play apart from the pitch if I’m being honest."  *(ID: 8, female, 41-50y, 1 child, Employed full-time)* |
| Reducing financial barriers | Increasing the availability of low-cost activities is viewed as important to reduce financial barriers to participation. | "I think you have to keep it very simple. I'll throw a random example out there like American football. It's really not an option because you need your helmet, you need your pads, you need all your protective equipment. So I think stay away from sports like that, keep it more simple so the likes of tennis. You know where you can get a big portable nest, things like that. Even kids, you know, taking your jumper off and going and setting them down for making goal posts. Ideas like rounders or cricket or simple things like that."  *(ID: 23, Male, 51-60 y, 1 child, Employed full-time)* |
|  | More transparency around costs and perceived value may support engagement by helping parents/guardians better understand and justify expenditure. | "I suppose if parents feel that it's worthwhile like activity, they don't mind paying a bit for it. I think they need to know what those costs are for and what are they paying for and how good of a bargain is it? Some people don't know, £15 is probably a bargain, but what is the real cost and do you know you're getting a bargain at £15. [It's] quite a lot of money, £15 for your child to kick a ball about so anybody could you could do that."  *(ID: 29, Female, 41-50y, 4 children, employed full-time)* |
|  | Providing discounts or subsidies is considered important to ensure affordability for those on a low-income while maintaining a sense of value and commitment. | "I don't necessarily think everything needs to be free. I think it's good for people to have to pay because then you will sort of take ownership of what you're doing...If you're paying something towards it, it gives you a sense of like, OK, there's a purpose for me to be here and do this. I'm putting some of my resources into this. But I do think there needs to be sort of a sliding scale for people you know. Some people are fine paying what they can pay for a sport, but then there's other people like myself that maybe, could you know benefit from having a reduced price?"  *(ID: 19, Female, 41-50 y, 4 children, Employed full-time)* |
| Increasing awareness, accessibility and inclusion | Limited and inconsistent provision for teenagers is identified as a key gap, highlighting the need for age-appropriate opportunities to support sustained engagement. | "Luckily enough, they have a summer scheme on for teenagers, which is only a £1 a day for one activity. So that's a good offer throughout July and August, for children in the local area. [For] their age group you find it harder to get soccer camps because the cut off is 13 or 14. I just find there's a loop in a lot of activities for, for, teenagers that are over 13 years. I've even seen in soccer, my sister sees it in Gaelic particularly over sixteens, to say 20 year olds, there's no teams for them children. I really think that should be something that should be encouraging particularly for mental health purposes."  *(ID: 10, Female, 51-60y, 3 children, Employed full-time)* |
|  | Improving accessibility of PA opportunities is viewed as essential to ensure equitable participation across different communities. | "To make it more easily accessible in terms of geography, maybe as well as price. It’s easy for me, I'm in Belfast and I'm not far from and parks and things like that. But for other people it's a distance probably. So it [PA opportunities] needs to be somewhere where they're gonna go anyway, and it's available and accessible."  *(ID: 18, Female, 51-60 y, 1 child, Employed part-time)* |
|  | Enhanced awareness and clearer signposting of available opportunities is needed to support engagement with existing provision. | "I think the simple things like signposting I think is very important for a start. It's kind of my job to plan things from my child. So I can take the time to do so but even then on a weekly basis, I'm checking out five different Facebook pages. I'm checking out council websites, private children's entertainment provision websites and then leisure centre websites to compile my list of opportunities and that's a lot of work. I'll speak to my family and they'll say...you're always got your ear to the ground on this kind of thing. But that's because I've got the time and I've developed kind of a network of where to look. So I think we really simple signposting a at one stop shop for people would make a big difference."  *(ID: 16, Other, 41-50 y, 1 child, Unemployed)* |
