# Supplementary material for: Free/low-cost physical activity opportunities for children and adolescents: Parental perspectives on participation, access and provision
Source: PLOS Glob Public Health. 2026 Nov 3;6(11):e0005770. doi: 10.1371/journal.pgph.0005770 (PMC13730368; doi:10.1371/journal.pgph.0005770)
Supplement: Supplementary file 8 [file pgph.0005770.s009.docx]

**Responses to Reviewer's Comments**

**Manuscript Title:** **Children’s participation in and access to free/low-cost physical activity opportunities in Northern Ireland (NI): parental perspectives.**

*We thank the Reviewer for their constructive and supportive comments. We have carefully considered each point and provide detailed responses in the table below. We believe these revisions have strengthened the manuscript, particularly in relation to methodological transparency and the integration of findings with the existing literature. We hope that it is now suitable for publication in PLOS ONE.*

|  | **Comment** | **Response** | **Line numbers** *‘Clean’ version of the manuscript* |
| --- | --- | --- | --- |
|  | ***Reviewer 1*** | | |
| ***Introduction*** | | | |
| **1.1** | Overall, the introduction is rather long and would benefit from being substantially shortened to achieve a clearer and more focused narrative. Several themes and topics are repeated across sections and could be consolidated into a single, more coherent paragraph. This would strengthen the conceptual clarity of the introduction and improve readability without losing essential context. | We have taken this comment on board and provided a more succinct introduction section which has improved readability without losing essential context. | 30-71 |
| **1.2** | For clarity, I recommend explicitly stating the age group for primary and post primary pupils, and work improving the readability of the sentence. | This has now been amended to read:  *“In Northern Ireland (NI), adherence to the guidelines is similarly low with only 21% of primary school children (10-12 years) and 16% of post-primary pupils (12-18 years) meeting current PA guidelines [6].”* | 37-39 |
| **1.3** | Line 41-42 The authors state that this phenomenon is well established in the literature; however, this claim is currently insufficiently substantiated. If the phenomenon is indeed well documented, the argument should be supported by additional and more comprehensive references. | Thank you for your comment. Based on an earlier comment to make the introduction more focused, we have removed this point around parental support and opted to mention it briefly alongside the barriers to PA among this population.  *‘’Some commonly cited barriers to PA among children and adolescents include a lack of fun, motivation, perceived lack of competence, body image, negative experiences, limited environmental opportunities and lack of support from family and friends [7–9].’’* | 39-42 |
| **1.4** | Line 53-55: It is unclear whether a change of 3% should be considered substantial in this context. The subsequent sentences appear to provide a stronger and more convincing justification for the authors’ argument. | We appreciate this suggestion and based on an earlier comment to make the introduction more focused, we have removed this point. |  |
| **1.5** | Line 59-67: This section would benefit from substantial rephrasing, as it is currently difficult to follow and appears conceptually cluttered. Clarifying the key message, improving the logical flow, and streamlining the argumentation would greatly enhance readability and strengthen the overall contribution of the paragraph. | This has now been amended to improve readability and avoid repetition:  *“Structured activities such as ‘Parkrun’, alongside more informal PA resources or opportunities including playparks, green spaces, recreational facilities and walkways have been associated with higher levels of PA [13,14]. However, access to such opportunities is often unequally distributed with disadvantaged communities having reduced availability of safe and accessible play spaces [15]. This is compounded by declining public investment in play provision for children across the United Kingdom (UK) [16,17].’’* | 52-58 |
| Materials and methods | | | |
| **2.1** | The quantitative component of the study appears insufficiently developed and somewhat uncertain in its current form. Methodological transparency is limited, particularly with regard to sample size and the handling of small subgroups. Reporting results as “n < 5” raises concerns about both statistical robustness and interpretability, as it complicates the assessment of the precision and reliability of estimates. A clearer justification of the quantitative approach, including sampling procedures, data handling, and analytic strategy, would strengthen the methodological rigor of the paper. | The purpose of the questionnaire has now been clarified as exploratory and descriptive within the mixed-methods design:  *“Given the exploratory nature of the questionnaire and the non-probability sampling approach, analyses were primarily descriptive.”*  We have expanded the description of the sampling approach and explicitly acknowledged the limitations associated with non-probability recruitment:  *“However, given the exploratory design and non-probability sampling approach, these findings should be interpreted as indicative of patterns rather than definitive group differences.”*  The limitations of this approach are also acknowledged in the discussion:  *“In addition as a non-probability, social media and snowball sampling approach was used, the sample may not be representative of the wider population and is subject to potential selection bias.”*  We have also clarified the rationale for the sample size calculation, framing it as a guiding benchmark rather than an indicator of representativeness:  *“Based on census data from 2021 that reported there were 1,098,200 adults aged 20–64 years living in NI, an a priori sample size calculation was performed. This indicated a minimum target of 663 participants (99%, α = 0.05) was required. This was used to ensure an adequate sample size was recruited, rather than to achieve population representativeness.”*  All analyses have been re-run with categories containing very small cell counts (n<5) excluded or combined, and this approach is now clearly stated in the methods:  *“To ensure statistical robustness, analyses were restricted to variables with adequate cell counts. Where any subgroup contained fewer than five observations, it was not included in the analysis.”*  In addition, the statistical analysis section has been refined to improve clarity and justification:  *“Data were analysed using SPSS v29 (IBM Corporation, New York, USA). Descriptive statistics are presented as means (standard deviations (SD)) for numerical data and counts (%) for categorical data. Given the exploratory nature of the questionnaire and the non-probability sampling approach, analyses were primarily descriptive.*  *For group comparisons, non-parametric tests were employed where appropriate due to non-normal data distributions. The Mann-Whitney U test was used for comparisons between two groups (for example household income; low vs high). The Kruskal Wallis test was used to compare between 3 or more means (for example index of multiple deprivation tertiles). For inferential analyses, categorical variables were assessed using Chi-square tests, with Fisher’s exact test applied where appropriate. To ensure statistical robustness, analyses were restricted to variables with adequate cell counts. Where any subgroup contained fewer than five observations, it was not included in the analysis. Statistical significance was set at p <.05; however, given the exploratory design and non-probability sampling approach, these findings should be interpreted as indicative of patterns rather than definitive group differences.”*  In addition, the result section has been revised to reflect the exploratory nature of the analyses:  *“Statistical significance was set at p<.05; however, given the exploratory design and non-probability sampling approach, these findings should be interpreted as indicative of patterns rather than definitive group differences.”* | 185-187  197-198  794-796  85-89  194-195  184-198  196-198 |
| **2.2** | Line 104-105: I would encourage the authors to provide a more detailed description of their inclusion criteria. At present, it remains unclear who was actually eligible to participate in the study, and whether, for example, older siblings could in principle also have responded on behalf of the target child. Clarifying these criteria (including who was invited, who was allowed to respond, and how potential ambiguities within families were handled) would considerably strengthen the transparency and interpretability of the findings. | This has been clarified within the manuscript:  *‘‘Eligibility criteria included being a parent/guardian aged ≥18 years of at least one child aged 4–16 years living in Northern Ireland (NI). The term ‘parent/guardian’ was intended to include any adult with a caregiving role for the child.”*  Recognising the diversity that exists in family structures, we were not prescriptive about the biological relationship between the parent/guardian and the child but those who self-identified as being a parent/guardian of a child aged 4-16 years, were eligible to participate.    Details of how participants were recruited/invited is addressed in lines 111-123. | 98-100 |
| **2.3** | Line 105: Have you considered allowing the older children to complete the questionnaire themselves rather than relying solely on proxy reports from parents? | Thank you for your comment. The focus of the current study was to explore parental/guardian perspectives on children’s engagement with free/low-cost physical activity opportunities. Parents/guardians are typically responsible for facilitating, financing, and supporting their children’s participation in such activities, and therefore were considered the most appropriate respondents for addressing the study aims.  We agree that including children and young people directly would provide important complementary insights, and this represents a valuable direction for future research but it beyond the scope of this project. |  |
| **2.4** | Line 111-118: as a reader one is left with the feeling that important information is missing. I would therefore encourage the authors to further develop and clarify this section. | Thank you for your comment. We have now addressed this by including additional information to provide further clarity on the process.  *“This study formed part of a larger research project, with full details of the questionnaire methodology published previously [12]. In brief, a cross-sectional online survey (Jisc; Bristol, UK) was administered between 11th March–29th May 2024. Based on census data from 2021 that reported there were 1,098,200 adults aged 20–64 years living in NI, an a priori sample size calculation was performed. This indicated that a minimum target of 663 participants (99%, α = 0.05) was required. This was used to ensure an adequate sample size was recruited, rather than to achieve population representativeness. Participants were recruited over a twelve-week period from March–June 2024, predominantly via social media (Facebook, X and Instagram) using a non-probability, snowball sampling approach, whereby participants shared the study within their networks. Parent groups, sports clubs, community organisations and public services (e.g. libraries) were also contacted to disseminate the survey link. As a non-probability sampling approach was used, the sample may not be representative of the wider population and findings should be interpreted with consideration of potential selection bias. No incentives were provided for participation.*  *Eligibility criteria included being a parent or guardian aged ≥18 years of at least one child aged 4–16 years living in Northern Ireland (NI). The term ‘parent/guardian’ was intended to include any adult with a caregiving role for the child. Screening questions were used to confirm eligibility prior to participation, and all respondents provided informed consent electronically. The survey was designed to take approximately 10 minutes to complete, and only fully completed responses were recorded on the Jisc platform. Those interested in taking part in the interviews provided contact details separately via MS Forms to preserve survey anonymity.*  *The questionnaire consisted of five sections: 1) demographic information and SES; 2) parental support for PA; 3) impact of the cost-of-living crisis; 4) impact of the cost-of-living crisis on crisis on access to sport and PA opportunities; 5) no/low- cost PA opportunities. The present study focuses on data from Section 5.”* | 83-108 |
| **2.5** | Line 121: Elaborate on which variables you are referring to here. | This has now been amended and more information added:  *‘’Data was also gathered on household income (before tax and deductions) [30], parents’ occupations, and the highest level of education attained by each parent.’’* | 116-118 |
| **2.6** | Line 127-130: Consider moving these lines up to the section where you describe the parents. | This has now been amended to make the section more coherent:  *“Parents/guardians were asked to self-report their gender and age. Based on the methodology of Donnelly et al. [21], several variables were collected to determine SES. Participants were asked to provide their home postcode which was entered into the NI Multiple Deprivation Measures [22] website and the deprivation score was recorded. Deprivation scores ranged from 0 to 890 and were divided into tertiles (Most deprived, 0-296; Average, 297-593; Least deprived, 594-890). From the NI Multiple Deprivation Measures system [22], local government area and urban, rural or mixed urban/rural classifications were extracted. Data was also gathered on household income (before tax and deductions) [23], parents’ occupations and the highest level of education attained by each parent. The median annual salary for full time employees in NI was £30,000 in 2022, which was used to categorise high and low household income. Information was also collected on benefit entitlement. Participants were asked to indicate the number of children aged 4-16 years that lived at home with them who they have regular responsibility for. Participants were also asked to indicate if they share the care of their children with a partner, ex-partner, grandparent or if they were a single parent [23].* | 110-123 |
| **2.7** | Line 130-134: Are these the SES variables you refer to earlier in the manuscript? If so, please harmonize the two sections. | This has now been amended to harmonize both sections and reads as follows:  “*Data was also gathered on household income (before tax and deductions) [23], parents’ occupations and the highest level of education attained by each parent. The median annual salary for full time employees in NI was £30,000 in 2022, which was used to categorise high and low household income. Information was also collected on benefit.’’* | 116-120 |
| **2.8** | Line 141: Please elaborate on why you have grouped "never" together with "once or twice a month" into the same category. There is a substantial difference between these response options, and I suggest considering three separate categories with " once or twice a year and never" as its own distinct group to improve clarity and analytical precision. | Thank you for your comment. We based this on the CSPPA methodology, which also takes a similar approach to the Health Behaviour in School-Age Children report (HBSC) (Elgar et al. 2023). Dichotomising or collapsing categories is widely accepted in behavioural or epidemiological research when the aim is to identify meaningful differences.  The rationale for this dichotomisation was to distinguish between children who engage in regular, habitual physical activity and those who participate infrequently or not at all. While we acknowledge that there are differences between response options such as “once or twice a month” and “never”, these were grouped to reflect a broader category of non-regular participation, which aligned with the primary aim of the analysis.  We agree that further stratification into additional categories could provide more granular insight; however, this would have resulted in smaller subgroup sizes and reduced statistical power. Therefore, a two-category approach was considered most appropriate for the current study. | 129-130 |
| **2.9** | Line 164-167: You fail to report whether this was actually achieved | This has now been amended:  *“The sample size (n=29) was considered sufficient to produce in-depth, rich accounts of lived experiences and to enable analytic generalisation, meaning the findings may be transferable to similar contexts rather than statistically representative of the broader population [25]. The sample demonstrated balanced representation across deprivation tertiles, and there was a sample from across the eleven council areas in NI.’’* | 150-155 |
| **2.10** | Line 171, line 185: Be consistent in whether you write "MS Team" or "Microsoft Teams" | This has been amended. | 160, 179,180 |
| **2.11** | Line 170: Remove the following text; PhD), a female academic with previous experience in qualitative research. It is not necessary. | This has been amended. |  |
| **2.12** | Line 175: Please elaborate on whether the same probe questions were used across all interviews or if new ones were developed each time. | This has now been amended and restructured to provide clarity:  *“The topic guide was used to ensure consistency across interviews while allowing flexibility for participants to elaborate on their experiences. Interviews began with brief demographic questions (e.g. employment status, annual household income, the ages of their children, and caregiving arrangements) followed by open-ended questions and prompts to explore responses in greater depth for example, “Why do you think this might be?”, “Can you tell us a little more about this?”.’* | 162-171 |
| **2.13** | Line 193-195: Could you please clarify what specific transformations were applied to the variables that violated normality assumptions, and provide more detail on how you determined that the transformed data still remained skewed in some cases? The current description is ambiguous and makes it difficult to assess the appropriateness of switching to non-parametric tests. For example, which variables were affected, what transformation methods (e.g., log, square root) were used, and what criteria (e.g., Shapiro-Wilk test post-transformation) led to the final choice of analytic approach? | *Thank you for this comment. In response to a previous comment, the Data Analysis section has been clarified and refined which we feel has provided additional transparency to the statistical analysis:*  *“Data were analysed using SPSS v29 (IBM Corporation, New York, USA). Descriptive statistics are presented as means (standard deviations (SD)) for numerical data and counts (%) for categorical data. Given the exploratory nature of the questionnaire and the non-probability sampling approach, analyses were primarily descriptive.*  *Data distributions were assessed for normality using the Shapiro–Wilk test alongside visual inspection of histograms and Q–Q plots. As several continuous variables were non-normally distributed, non-parametric tests were used for group comparisons. The Mann-Whitney U test was used for comparisons between two groups (for example household income; low vs high). The Kruskal Wallis test was used to compare between 3 or more means (for example index of multiple deprivation tertiles). For inferential analyses, categorical variables were assessed using Chi-square tests, with Fisher’s exact test applied where appropriate. To ensure statistical robustness, analyses were restricted to variables with adequate cell counts. Where any subgroup contained fewer than five observations, it was not included in the analysis. Statistical significance was set at p <.05; however, given the exploratory design and non-probability sampling approach, these findings should be interpreted as indicative of patterns rather than definitive group differences.”* | 184-198 |
| **2.14** | Line 200-202: Please elaborate on what additional information is being collected here? | This has now been amended to read:  *‘’Components of the online questionnaire had built-in, optional answer probes so that further information could be obtained to contextualize data on participants experiences.’’* | 201-202 |
| **Results** | | | |
| **3.1** | Overall, the section on questionnaire results requires substantial revision for clarity, structure, and completeness. I recommend thoroughly rewriting it, drawing inspiration from how similar studies have presented their findings—both in terms of writing style and the specific details reported (e.g., response rates, descriptive statistics by subgroup, effect sizes, and clear links to research questions). Additionally, there is a missing "=" between "n" and the number in several places throughout the text. | The questionnaire results section has been revised to improve clarity, structure, and consistency of reporting.  Specifically, we have:   - Refined the overall structure and flow of the section to improve readability. - Corrected formatting inconsistencies (e.g., use of “n=” throughout). - Ensured clearer linkage between results and the study aims.   Given the exploratory nature of the study and the non-probability sampling approach, the focus of the quantitative analysis was primarily descriptive. Therefore, effect sizes and response rates were not included, as these were not considered appropriate for the study design. |  |
| **3.2** | Line 224: How many individuals were invited to participate in the questionnaire, and do you have a response rate? This would help indicate whether the 885 participants are representative or not. | Thank you for your comment. As participants were recruited via a non-probability, social media–based snowball sampling approach, it was not possible to determine the total number of individuals who were exposed to or invited to participate in the questionnaire. Consequently, a response rate could not be calculated. However, we have clarified the recruitment strategy within the manuscript:  *“Participants were recruited over a twelve-week period from March–June 2024, predominantly via social media (Facebook, X and Instagram) using a non-probability, snowball sampling approach, whereby participants shared the study with friends and family. Parent groups, sports clubs, community organisations and public services (e.g. libraries) were also contacted to disseminate the link to the questionnaire’’*  We have also clarified that the sample size calculation was used as a guiding benchmark rather than an indicator of representativeness, and have explicitly acknowledged in the discussion that the sample may not be representative of the wider population due to the sampling approach:  “*Based on census data from 2021 that reported there were 1,098,200 adults aged 20–64 years living in NI, an a priori sample size calculation was performed. This indicated a minimum target of 663 participants (99%, α = 0.05) was required. This was used to ensure an adequate sample size was recruited, rather than to achieve population representativeness.”* | 89-93  86-89 |
| **3.3** | Line 229-232: Avoid using "majority" in two consecutive sentences; please rephrase for improved variety in wording. | This has been amended to read:  *‘’Among those in employment, the largest proportion were in Professional Occupations (32.8%)’’* | 248-249 |
| **3.4** | Line 236 (Table 1): The table appears to describe the participation process, but this is unclear without further context from the main text. Tables should be fully self-explanatory and interpretable independently of the manuscript body, with comprehensive footnotes, clear headings, and defined abbreviations to ensure standalone readability. Additionally, quantitative data for subgroups with fewer than 5 individuals should not be reported, as it compromises data anonymity, makes the dataset overly identifiable, and undermines the strength and robustness of the data. | Table 1 has been revised to improve clarity, standalone interpretability, and consistency of reporting.  Categories with very small cell counts have been combined or suppressed where appropriate, particularly within the interview sample, to protect participant confidentiality. In a small number of cases where cell counts remain low, these have been retained where the risk of identification is considered minimal and where further aggregation would reduce interpretability. | 255 |
| **3.5** | Line 251-253: Rephrase to make it easier to understand. | This has been amended to read better throughout the whole paragraph*:*  *“Almost all parents/guardians (99.3%) indicated that their children took part in at least one free/low-cost PA opportunity at least once per week. The majority of parents/guardians reported that their children were walking (91.3%) or attending an outdoor playpark (63.7%). Half of parents/guardians reported that their children were exercising at home (57.7%), cycling (51.9%) or going to council or community pitches (52.3%). Outdoor gyms were reported to be used infrequently by parents/guardians with only 9.8% of parents/guardians reporting that their children used these facilities at least once per week.”* | 263-269 |
| **3.6** | Line 256-259: The parenthetical statements are confusing for readers and obscure the intended meaning. Please rephrase to better assist readers in understanding the connection and flow without requiring additional effort to parse. | This has been amended, please see response to Point 3.5 above. |  |
| **3.7** | Line 270-276: You are missing your own data/numbers—what do they show? This is the information you should present here. | This has been amended with the addition of the correlating p values:  *“No statistically significant differences in perceived access to free/low-cost PA opportunities were detected based on parental support for PA (P=0.206) (S3 Table). There was some evidence of differences in perceived access based benefit entitlement (P=<0.001) (S4 Table). However, across other indicators of socioeconomic status, including household income, index of multiple deprivation, family structure, and employment status, no statistically significant differences were observed.”* | 285-290 |
| Interviews | | | |
| **4.1** | Consider removing the overview section or incorporating direct quotes into it to substantiate the claims, then deleting the subsequent section. Generally, the current approach is thin, as each claim is supported by only a single quote despite having 29 respondents | Taking this valuable comment on board, we have revised the interview results section to strengthen the depth of analysis. Specifically, we revisited the thematic analysis to refine theme and sub-theme descriptions, strengthen analytical interpretation, and ensure that each theme is supported by a broader range of illustrative quotations to better represent the diversity of participant perspectives. We believe these revisions have enhanced the analytical depth and overall robustness of the findings. |  |
| **4.2** | Line 284: Did you achieve the target of 10 participants from each tertile? If not, please clarify. If so, did they represent 10 from each group? I could not find this information in Table 2. | This has been amended to reflect this comment:  *“The sample demonstrated balanced representation across deprivation tertiles (most deprived n=10, average deprivation (n=9) and least deprived n=10), and there was representation across all eleven local council areas in NI.”*  In addition, the distribution of participants across deprivation tertiles is outlined in Table 1. | 153-155 |
| **4.3** | Line 285: MS Teams (n=21; 72%) and the mean interview duration was 29 (SD =9) | This text has been deleted as it was previously reported in the methodology. | 180 |
| **Discussion** | | | |
| **5.1** | The discussion largely repeats the results without engaging substantively with the existing literature. It should instead address whether your findings align with or contradict prior research, highlighting consistencies, discrepancies, and their implications for the field. | Thank you for this important comment. In response, we have substantially revised the Discussion section to reduce repetition of the Results and strengthen engagement with the existing literature. Rather than restating findings, we now focus on interpreting the results in the context of prior research.  We have also strengthened discussion of key subgroups identified in the qualitative data (e.g., adolescents, girls, and children with additional needs) by linking these findings to established literature on age- and gender-related differences in physical activity and known barriers to participation.  Overall, we believe these revisions have enhanced the analytical depth of the Discussion and ensured that our findings are more clearly situated within the broader evidence base, while also highlighting their implications for policy and practice. |  |
| **5.2** | Further the manuscript does not currently address the very wide age range of the children about whom parents are reporting. As a reader, it is impossible to discern whether the majority of the sample consists of younger or older children, which may substantially influence how parents perceive and respond to questions about needs and use of free services. It seems likely that the needs and relevant offers for a 4-year-old differ markedly from those of a 16-year-old, and this heterogeneity may have important implications for the interpretation of the findings. I would therefore encourage the authors to describe and reflect more explicitly on the age distribution of the child sample and to consider whether, and how, age might shape parents’ responses and the overall conclusions of the study | This has now been added as a limitation:  *“The inclusion criteria for this study encompassed parents/guardians of children aged 4–16 years, resulting in a wide age range. This may represent a limitation, as PA preferences, and opportunities differ substantially between younger children and older adolescents. However, existing evidence suggests that participation in organised sport begins in early childhood and varies by socioeconomic status [63,64]. Including parents of younger children therefore enabled exploration of participation in free/low-cost PA opportunities across developmental stages. Future research may benefit from stratifying findings by age group to better understand age-specific needs and patterns of participation.’’*  Furthermore, within the questionnaire results it is stated that:  “*On average, the children were aged 10.1 years (SD 3.8).”*  Within the interview results, it is outlined that:  *“In total, n=29 parents/guardians of n=54 children aged 4-16 years (mean age: 10.3 (SD 4.4) years) participated in the interviews.”* | 800-807  246  304-305 |
| **5.3** | Line 473-474: Can your data support making claims about this? | We appreciate that this may have read like an overstatement and it has now been amended with additional literature:  *‘’Interestingly, within the present study there were no differences in participation in free/low-cost PA opportunities by indicators of SES. This was somewhat unexpected given that previous research reports higher PA participation and a greater likelihood of meeting PA guidelines among individuals of higher SES [12]. One possible explanation for this is that the ongoing cost-of-living crisis may have encouraged families across all socioeconomic backgrounds to rely more heavily on free/low-cost opportunities as substitutes for structured, paid activities [33], rather than an absence of underlying inequalities. Consistent with this, it has been previously reported that among parents/guardians in NI, 29% of parents/guardians said their children have started/increased the number of free activities that they do (such as walking and running) [10]. ‘’* | 696-704 |
| **5.4** | Line 485-487: Here, you contradict yourselves, as you have just stated earlier that there was no difference according to your data. | This has been amended to ensure there is no contradiction:  ***“****One possible solution to low levels of awareness is an accessible, centralised platform that provides up-to-date, localised information on free/low-cost PA opportunities for children, adolescents, and families. Such an approach may help reduce inequalities by improving visibility and supporting parental decision-making as greater awareness of community services for PA has been shown to support planning and engagement [57].”* | 761-765 |
| **5.5** | Line 488-496(586): All of this is a repetition of your results section. This should be rewritten to instead discuss what the literature says about this topic in relation to your findings—does it support them, or is there disagreement? | Thank you for your comment. This section has now been amended/restructured to address these shortcomings:  *“Despite high levels of participation in free/low-cost PA opportunities, the majority of parents/guardians in NI (76%) perceived there to be insufficient provision for children and adolescents in their local area. Within this research, gaps in provision were evident for particular groups including adolescents, girls and children with additional needs. These findings align with existing literature demonstrating that girls engage in less PA than boys and are less likely to meet recommended guidelines [5,34]. Furthermore, PA levels decline with age, with evidence showing substantial reductions during adolescence, particularly among girls [35–39]. This reinforces the importance of targeting adolescent populations, especially girls, as a public health priority. Given that PA behaviours established during adolescence often track into adulthood and are associated with long-term health outcomes [40–42], addressing these gaps in provision may be critical for improving population health. Similarly, children and adolescents with disabilities or additional needs are consistently reported to be less physically active than their peers and face additional barriers including limited access to appropriate facilities, higher costs, transport challenges, and a lack of tailored provision [43,44]. Addressing these disparities requires tailored, inclusive approaches that meet the needs of all children and adolescents.’’*  The point around understanding local provision has also been strengthened:  *“While no differences in participation in free/low-cost PA opportunities based on index of multiple deprivation were observed within the current study, there is a need to profile and better understand the availability of such opportunities across communities in NI, particularly as evidence from other UK regions shows that the availability of PA facilities generally declines as levels of deprivation increase [48].’’* | 705-719  730-734 |
| **5.6** | Line 499-500 (602-603): A reference is missing here. | This has now been included. 48. Farrell L, Hollingsworth B, Propper C, Shields MA. The socioeconomic gradient in physical inactivity: Evidence from one million adults in England. Soc Sci Med. 2014;123:55–63. | 734 |
| **5.7** | Line 506-520: All of this is a repetition of your results section. This should be rewritten to instead discuss what the literature says about this topic in relation to your findings—does it support them, or is there disagreement? | This has now been amended and rewritten in parts to reflect the reviewer’s comment and ensure that it is a discussion of findings:  *“Parents/guardians emphasised the importance of long-term investment, sustainable infrastructure and policy change to improve access to PA opportunities for children and adolescents. These perceptions align with national and international research and policy frameworks advocating for sustained investment in PA promotion [56,58,59].*  *In addition, while parents/guardians were cognisant of the challenges this would bring, they suggested targeted supports for low-income families, such as means-tested subsidies as low-income families are frequently described as a ‘hard to reach’ population [60]. Children from these households are less likely to engage in regular PA, [6,11,12] and face additional structural barriers. These findings highlight the importance of equitable and inclusive provision.’’* | 768-777 |
| **5.8** | Line 548-549 (conclusion) : Be cautious not to draw conclusions that your own data cannot support. | This has now been removed and the conclusion has been rewritten:  *“Despite high levels of engagement in free/low-cost PA opportunities, this study highlights important gaps in provision, accessibility and awareness across NI. While informal activities such as walking and outdoor play were popular, most parents perceived there to be insufficient provision for children and adolescents within their local area. Structural issues including limited local availability, poor facilities and low awareness were identified as barriers to participation, particularly for adolescents, girls and children with additional needs.*  *Findings from this mixed-methods study suggest that improving participation in free/low-cost PA opportunities is a complex issue which requires more than increasing provision alone. A coordinated approach is needed which encompasses a more extensive range of provision, improved facilities, enhanced affordability and targeted financial support for families experiencing socioeconomic disadvantage.*  *Free/low-cost PA opportunities represent a valuable and scalable strategy to support children’s PA and may play a critical role in reducing inequalities, particularly within the context of the ongoing cost-of-living crisis.”* | 817-831 |
| **5.9** | Line 551: I would encourage the authors to use a different term instead of "whole-system approach," as it has not been mentioned previously in the manuscript and therefore appears confusing when suddenly introduced in the conclusion. | Similar to the comment above, this has been removed and the conclusion has been rewritten. | 817-831 |
