# Supplementary material for: Free/low-cost physical activity opportunities for children and adolescents: Parental perspectives on participation, access and provision
Source: PLOS Glob Public Health. 2026 Nov 3;6(11):e0005770. doi: 10.1371/journal.pgph.0005770 (PMC13730368; doi:10.1371/journal.pgph.0005770)
Supplement: Supplementary file 9 [file pgph.0005770.s010.docx]

**Reviewer's Responses to Questions**

**Manuscript ID:** PGPH-D-25-03876R1

**Manuscript Title:** Free/low-cost physical activity opportunities for children and adolescents: parental perspectives on participation, access and provision.

*We sincerely thank the reviewer for their thoughtful, insightful and constructive comments. We are grateful for the time taken to highlight the strengths of the study and manuscript, and we appreciate the positive feedback. We have carefully considered and addressed each of the reviewer’s suggestions in the revised manuscript and provide a point-by-point response below. Please note that the line numbers referenced throughout this rebuttal correspond to the clean version of the revised manuscript.*

|  | **Comment** | **Response** | **Line numbers**  (on clean manuscript) |
| --- | --- | --- | --- |
| **Abstract** | | | |
| 1 | The abstracts generally remain at the level of descriptive summaries, and the theoretical contribution of the research is not sufficiently revealed. Since the current abstracts closely follow a structure of “problem exists → investigation → policy need,” they may feel somewhat lacking in novelty by the standards of Plos Global Public Health journals. The analysis is also presented primarily based on simple frequency results, appearing to lack analytical depth. In particular, the lack of a theoretical explanation regarding “why inequalities persist” in the abstract risks reading it as a simple policy report. | We have revised the abstract to strengthen the research gap and move beyond a descriptive summary of the findings. In particular, the revised abstract now highlights the multiple factors beyond cost that influence access to and engagement with free/low-cost PA opportunities and better reflects the mixed-methods findings. We have also refined the title to make the knowledge contribution clearer: “Free/low-cost physical activity opportunities for children and adolescents: parental perspectives on participation, access and provision.” | 1-36 |
| **Introduction** | | | |
| 2 | The biggest weakness of the introduction is the absence of a clear theoretical framework. Currently, the introduction lists various prior studies, but there is no central theory to integrate them. For example, the academic completeness would have been much higher if the research problem had been structured using the socioecological model, the environmental justice framework, or health equity theory. | We appreciate this comment and agree that the theoretical framing of the manuscript could be strengthened, particularly given that the theoretical contribution has emerged more clearly from the findings. We considered several established frameworks and feel that this research aligns most closely with the Health Equity Framework (HEF), given its focus on equity and fair access to resources and opportunities and the interaction between factors operating at multiple levels. We have therefore used the HEF as a conceptual lens to strengthen the framing and interpretation of the findings, and have added the following to the manuscript:  Introduction: “Access to PA opportunities is influenced by a range of factors operating across multiple and interacting levels. The Health Equity Framework provides a useful lens for understanding these influences, placing equity and fair access to resources and opportunities at the centre of health and wellbeing and recognising the interaction between individual, social, environmental and structural factors [18].  While emerging evidence suggests that removing financial barriers can increase PA participation and may help to reduce inequalities, findings are mixed, particularly for children and adolescents [19]. A recent scoping review concluded that while subsidised or free opportunities can improve participation and engage children from lower socioeconomic backgrounds, the impact of these initiatives decreases with increasing deprivation [20]. Research suggests that free/low-cost activities (i.e. those with no participation fee/membership or specialised equipment required) alone may not encourage those living in disadvantaged neighbourhoods to take up regular PA [21]. This suggests that additional factors may influence whether children are able to access and benefit from free or low-cost provision. However, existing research has largely focused on whether free or subsidised opportunities increase participation, with less attention given to how families experience these opportunities and the factors that may facilitate or constrain access. Understanding these factors is important for informing strategies to promote equitable PA participation.”  Methods: “The Health Equity Framework [18] was used as a conceptual lens to interpret the findings.”  Discussion: “The findings can be understood through the Health Equity Framework [18], which emphasises the importance of fair access to resources and opportunities for health and recognises that health is influenced by interacting factors across multiple levels.”  “While free/low-cost opportunities may reduce financial barriers to PA, our findings suggest that financial cost is only one aspect of access with location, transport, availability and quality of opportunities also influencing participation. It is this interaction of individual, social and environmental factors that appears to influence whether opportunities are accessible and suitable for families.  Despite high levels of participation in free/low-cost PA opportunities, the majority of parents/guardians in NI (76%) perceived there to be insufficient provision for children and adolescents in their local area. This discrepancy between high levels of participation and perceptions of insufficient provision highlights that participation in some free/low-cost activities does not necessarily indicate equitable access to a sufficient range of opportunities.”  Conclusion: “Findings from this mixed-methods study suggest that improving participation in free/low-cost PA opportunities is a complex issue which requires more than increasing provision alone. Free/low-cost provision may reduce financial barriers to participation, but our findings suggest that access is also influenced by factors including local availability, transport, quality, suitability and awareness of opportunities. These findings highlight the importance of considering the multiple and interacting factors that influence access to and participation in free/low-cost PA opportunities. Therefore, a coordinated approach is needed which encompasses a more extensive range of provision, improved facilities, enhanced affordability and targeted financial support for families experiencing socioeconomic disadvantage.  Free/low-cost PA opportunities represent a valuable and potentially scalable strategy to support children’s PA, particularly within the context of the ongoing cost-of-living crisis. However, their potential to support equitable PA participation depends not only on whether opportunities are free or affordable, but also on whether they are accessible, suitable and known to families.” | 65-82  251  632-634  650-659  768-780 |
| 3 | The research gap is also somewhat weak. Although the phrase “evidence is limited” is repeated, there is a lack of explanation regarding the conceptual gap—specifically what existing research failed to explain. Consequently, while the introduction appears like a well-organized public health report, it lacks the strong theoretical positioning required by Plos Global Public Health. | We have strengthened the theoretical framing of the introduction and clarified the conceptual gap addressed by the study. Specifically, we have clarified that, although existing research has examined whether free or subsidised opportunities increase participation, less is known about families' experiences of these opportunities and the factors that facilitate or constrain access. This gap is directly addressed by our mixed-methods study, which examines parental perspectives on engagement, participation, local availability and perceived barriers to free/low-cost PA opportunities:  “While emerging evidence suggests that removing financial barriers can increase PA participation and may help to reduce inequalities, findings are mixed, particularly for children and adolescents [19]. A recent scoping review concluded that while subsidised or free opportunities can improve participation and engage children from lower socioeconomic backgrounds, the impact of these initiatives decreases with increasing deprivation [20]. Research suggests that free/low-cost activities (i.e. those with no participation fee/membership or specialised equipment required) alone may not encourage those living in disadvantaged neighbourhoods to take up regular PA [21]. This suggests that additional factors may influence whether children are able to access and benefit from free or low-cost provision. However, existing research has largely focused on whether free or subsidised opportunities increase participation, with less attention given to how families experience these opportunities and the factors that may facilitate or constrain access. Understanding these factors is important for informing strategies to promote equitable PA participation” | 70-82 |
| **Methods** | | | |
| 4 | Quantitative analysis at the Plos Global Public Health level is somewhat simple. It is mostly centered on frequency analysis and group comparison, and lacks in-depth analyses such as regression, mediation, moderation, and multilevel analysis. It is regrettable that an explanatory model is absent, despite the research topic itself dealing with inequality and access. | We agree that more complex statistical modelling can provide additional insight into relationships between variables; however, such analyses were outside the scope of the current study. The aim of this study was to explore parental perspectives on children’s engagement with free/low-cost PA opportunities, factors affecting participation, local availability and views on future provision. Given the exploratory nature of the questionnaire and the non-probability sampling approach, the quantitative analysis was primarily descriptive, with between-group comparisons used to examine differences in participation and perceptions of access across various indicators of socioeconomic status. The quantitative component was intended to characterise patterns within the sample and complement the qualitative exploration of parents’ experiences, rather than to develop a causal or explanatory model of inequalities in PA participation. |  |
| 5 | Furthermore, since all PA variables and access perception are based on self-reports, there is a high possibility of measurement bias. | We agree that the use of self-reported measures may introduce measurement and recall bias. This has been acknowledged as a limitation in the Discussion:  “In addition, frequency of engagement in free/low-cost PA opportunities was self-reported by parents/guardians and may be subject to measurement or recall bias.” | 752-754 |
| 6 | Another limitation is the exclusion of objective environmental data (GIS, facility audits, accelerometer data). | The inclusion of objective measures of PA and the local environment could have provided additional information to complement the self-reported data. However, these measures were outside the scope of the current study. We have acknowledged this as a limitation and identified their inclusion as an area for future research:  “In addition, frequency of engagement in free/low-cost PA opportunities was self-reported by parents/guardians and may be subject to measurement or recall bias. Future research should consider the inclusion of device-measured PA and objective environmental data, such as Geographic Information Systems (GIS) based assessment of local facilities and opportunities.” | 752-756 |
| 7 | Additionally, snowball sampling and social media recruitment methods can lead to representativeness issues, and a significant methodological limitation is that the sample is actually biased towards women, the highly educated, and the high-income bracket. | We have strengthened the limitations section to explicitly acknowledge the demographic characteristics of the sample, including the predominance of female participants and those in employment, as well as the non-probability sampling approach. We have also clarified that the findings may represent a ‘better-case scenario’ and highlighted the need for further research with low-income families:  “While participants were purposively sampled across deprivation tertiles to ensure socioeconomic diversity, it must be acknowledged that for both the survey and the interviews the majority of participants were female and in employment, with the majority having a higher level of education. In addition, as a non-probability, social media and snowball sampling approach was used, the sample may not be representative of the wider population and is subject to potential selection bias.” | 733-739 |
| **Results** | | | |
| 8 | The results remain at an overall descriptive level. Most of the outcomes are centered on frequency and perception, and there are few statistical models explaining the relationships between variables. | This point is addressed in our response to Comment 4. As outlined above, the quantitative component was designed to characterise patterns within the sample and examine between-group differences, rather than to develop a causal or explanatory statistical model. This is outlined in the methods. |  |
| 9 | In particular, the finding that “SES differences were not significant” could have been an interesting discovery, but further analysis on it is lacking. For example, the results would have been much stronger if interaction effects or subgroup analysis had been included. | We have strengthened the results and discussion to highlight that, while no statistically significant differences in participation were observed across most socioeconomic indicators, participation differed significantly by education level (p=0.024). We have interpreted this finding cautiously given that the pattern did not represent a clear socioeconomic gradient. We have also considered possible explanations for the absence of significant differences across most socioeconomic indicators, including the possibility that families across socioeconomic groups may have increased their reliance on free/low-cost activities during the cost-of-living crisis:  “As outlined in Supplementary Table 2 (S2 Table), no statistically significant differences in participation in free/low-cost PA opportunities were observed across most indicators of SES including household income, index of multiple deprivation, benefit entitlement, employment status or between those residing in urban, rural or mixed urban/rural areas (P>0.05). However, participation differed significantly by education level (P=0.024), although there was no clear socioeconomic gradient.”  “Interestingly, within the present study there were no differences in participation in free/low-cost PA opportunities by most indicators of SES, including household income and deprivation tertile. Although there were differences based on level of education (P=0.024), the results did not show a clear socioeconomic gradient. These results were somewhat unexpected given that previous research reports higher PA participation and a greater likelihood of meeting PA guidelines among individuals of higher SES [12]. One possible explanation for this is that the ongoing cost-of-living crisis may have encouraged families across all socioeconomic backgrounds to rely more heavily on free/low-cost opportunities as substitutes for structured, paid activities [34], rather than an absence of underlying inequalities. Consistent with this, it has been previously reported that among parents/guardians in NI, 29% of parents/guardians said their children have started/increased the number of free activities that they do (such as walking and running) [35,36]” | 289-294  639-650 |
| 10 | Additionally, while the qualitative findings are rich, the conceptual integration between themes is somewhat weak, and some results are presented repeatedly, which may feel a bit verbose. This reviewer considers it to be “interesting data but limited analytical depth.” | We have undertaken substantial revisions to the Results section to improve the structure and analytical coherence of the qualitative findings. The interview findings are now organised around two overarching themes: (1) What is currently available and who can access it? and (2) What do parents think needs to change? Findings have been consolidated to reduce repetition and greater emphasis has been placed on the factors that interact to influence access to and engagement with free/low-cost PA opportunities. These revisions also provide stronger alignment with the Health Equity Framework, which is used as a conceptual lens to interpret the findings. We have also further strengthened this conceptual integration through the addition of a figure illustrating the relationship between the themes and the HEF. | 252-617 |
| **Discussion** | | | |
| 11 | The discussion also lacks theoretical contribution. Current discussions are mostly centered on "explanation of results + policy proposals" and lack broader conceptual implications. In particular, there is a lack of a deeper sociological interpretation regarding why free/low-cost opportunities do not sufficiently resolve inequality. | We have strengthened the theoretical contribution throughout the Discussion by applying the Health Equity Framework to interpret the findings and consider the wider factors influencing equitable access to free/low-cost PA opportunities. In particular, we highlight that financial cost is only one aspect of access, with the interaction of individual, social and environmental factors also influencing whether opportunities are accessible and suitable for families. We further consider how the organisation, funding, promotion and distribution of opportunities may influence access, and emphasise that universally available free provision will not necessarily result in equitable participation.  “The findings can be understood through the Health Equity Framework [18], which emphasises the importance of fair access to resources and opportunities for health and recognises that health is influenced by interacting factors across multiple levels.”  “While free/low-cost opportunities may reduce financial barriers to PA, our findings suggest that financial cost is only one aspect of access with location, transport, availability and quality of opportunities also influencing participation. It is this interaction of individual, social and environmental factors that appears to influence whether opportunities are accessible and suitable for families.”  “Looking through the lens of the Health Equity Framework [18], in particular the systems of power, it would appear the issue isn’t simply that parents fail to find activities. Rather, the way opportunities are organised, funded, promoted and distributed influences whether families can access them. From a health equity perspective, improving awareness represents an important but insufficient intervention if looked at in isolation. A centralised platform may support parents' role as gatekeepers to children’s PA, but equitable participation also requires provision that is affordable, flexible, locally available and responsive to children's interests. Sustained investment, infrastructure and policy support are therefore necessary to address the structural conditions that shape families' opportunities to engage in PA [59,60]”  “Children from these households are less likely to engage in regular PA, [6,11,12] and may face additional barriers to participation, included limited access to suitable and affordable opportunities. These findings highlight the importance of equitable and inclusive provision, rather than assuming that universally available free provision will necessarily produce equitable participation.” | 632-634  650-654  707-715  718-722 |
| 12 | Additionally, the latter part of the discussion is somewhat repetitive, and some sentences give the impression of simply repeating the results. | We thank the reviewer for this comment. We have reviewed the discussion for repetition and edited the text to remove duplication and ensure it is concise. |  |
| **Conclusion** | | | |
| 13 | The conclusion remains somewhat predictable. While the message that “more support and accessibility are needed” is valid, stronger theoretical or empirical takeaways may be required by top-tier SSCI standards. | We have substantially revised the conclusion to strengthen the conceptual contribution of the study and highlight that affordability alone does not ensure equitable access, with wider factors influencing access and engagement.  “Despite high levels of engagement in free/low-cost PA opportunities, this study highlights important gaps in provision, accessibility and awareness. While informal activities such as walking and outdoor play were popular, most parents perceived there to be insufficient provision for children and adolescents within their local area. Structural issues including limited local availability, poor facilities and low awareness were identified as barriers to participation, particularly for adolescents, girls and children with additional needs.  Findings from this mixed-methods study suggest that improving participation in free/low-cost PA opportunities is a complex issue which requires more than increasing provision alone. Free/low-cost provision may reduce financial barriers to participation, but our findings suggest that access is also influenced by factors including local availability, transport, quality, suitability and awareness of opportunities. These findings highlight the importance of considering the multiple and interacting factors that influence access to and participation in free/low-cost PA opportunities. Therefore, a coordinated approach is needed which encompasses a more extensive range of provision, improved facilities, enhanced affordability and targeted financial support for families experiencing socioeconomic disadvantage.  Free/low-cost PA opportunities represent a valuable and potentially scalable strategy to support children’s PA, particularly within the context of the ongoing cost-of-living crisis. However, their potential to support equitable PA participation depends not only on whether opportunities are free or affordable, but also on whether they are accessible, suitable and known to families.” | 762-780 |
| 14 | Furthermore, the lack of a final message emphasizing the research's originality means that the new insights provided to the existing literature are conveyed relatively weakly. | We have revised the final paragraph to highlight the key contribution of the study: that while free/low-cost PA opportunities represent a valuable and potentially scalable strategy to support children’s PA, equitable access depends on wider factors influencing access and participation.  “Free/low-cost PA opportunities represent a valuable and potentially scalable strategy to support children’s PA, particularly within the context of the ongoing cost-of-living crisis. However, their potential to support equitable PA participation depends not only on whether opportunities are free or affordable, but also on whether they are accessible, suitable and known to families.” | 777-780 |
