# Supplementary material for: Free/low-cost physical activity opportunities for children and adolescents: Parental perspectives on participation, access and provision
Source: PLOS Glob Public Health. 2026 Nov 3;6(11):e0005770. doi: 10.1371/journal.pgph.0005770 (PMC13730368; doi:10.1371/journal.pgph.0005770)
Supplement: Peer review history

# Peer review: Decision Letter 0

**DOI:** 10.1371/journal.pgph.0005770.r001

**Authors:** Jennifer Tucker

15 Feb 2026

PGPH-D-25-03876

Children’s participation in and access to free/low-cost physical activity opportunities in Northern Ireland (NI): the parent's perspective.

PLOS Global Public Health

Dear Dr. McCafferty,

Thank you for submitting your manuscript to PLOS Global Public Health. After careful consideration, we feel that it has merit but does not fully meet PLOS Global Public Health’s publication criteria as it currently stands. Therefore, we invite you to submit a revised version of the manuscript that addresses the points raised during the review process.

Please note that we have only been able to secure a single reviewer to assess your manuscript. We are issuing a decision on your manuscript at this point to prevent further delays in the evaluation of your manuscript. Please be aware that the editor who handles your revised manuscript might find it necessary to invite additional reviewers to assess this work once the revised manuscript is submitted. However, we will aim to proceed on the basis of this single review if possible.

Please submit your revised manuscript by Mar 30 2026 11:59PM. If you will need more time than this to complete your revisions, please reply to this message or contact the journal office at globalpubhealth@plos.org. When you're ready to submit your revision, log on to https://www.editorialmanager.com/pgph/ and select the 'Submissions Needing Revision' folder to locate your manuscript file.

Please include the following items when submitting your revised manuscript:

- A letter that responds to each point raised by the editor and reviewer(s). You should upload this letter as a separate file labeled 'Response to Reviewers'.
- A marked-up copy of your manuscript that highlights changes made to the original version. You should upload this as a separate file labeled 'Revised Manuscript with Track Changes'.
- An unmarked version of your revised paper without tracked changes. You should upload this as a separate file labeled 'Manuscript'.

Guidelines for resubmitting your figure files are available below the reviewer comments at the end of this letter.

We look forward to receiving your revised manuscript.

Kind regards,

Jennifer Tucker, PhD

Staff Editor

PLOS Global Public Health

Journal Requirements:

1. Please amend your detailed Financial Disclosure statement. This is published with the article. It must therefore be completed in full sentences and contain the exact wording you wish to be published.

i. Please clarify all sources of financial support for your study. List the grants, grant numbers, and organizations that funded your study, including funding received from your institution. Please note that suppliers of material support, including research materials, should be recognized in the Acknowledgements section rather than in the Financial Disclosure.

ii. State the initials, alongside each funding source, of each author to receive each grant. For example: "This work was supported by the National Institutes of Health (####### to AM; ###### to CJ) and the National Science Foundation (###### to AM)."

iii. State what role the funders took in the study. If the funders had no role in your study, please state: “The funders had no role in study design, data collection and analysis, decision to publish, or preparation of the manuscript.”

iv. If any authors received a salary from any of your funders, please state which authors and which funders.

2. Please ensure that your Ethics Statement is available in its entirety at the beginning of your Methods section, under a subheading 'Ethics Statement'.

3. We have noticed that you have uploaded Supporting Information files, but you have not included a list of legends. Please add a full list of legends for your Supporting Information files after the references list.

4. In the online submission form, you indicated that “The datasets used and/or analysed during the current study are available from the corresponding author on reasonable request.”.

All PLOS journals now require all data underlying the findings described in their manuscript to be freely available to other researchers, either

1. In a public repository,

2. Within the manuscript itself, or

3. Uploaded as supplementary information.

This policy applies to all data except where public deposition would breach compliance with the protocol approved by your research ethics board. If your data cannot be made publicly available for ethical or legal reasons (e.g., public availability would compromise patient privacy), please explain your reasons by return email and your exemption request will be escalated to the editor for approval. Your exemption request will be handled independently and will not hold up the peer review process, but will need to be resolved should your manuscript be accepted for publication. One of the Editorial team will then be in touch if there are any issues.

If the reviewer comments include a recommendation to cite specific previously published works, please review and evaluate these publications to determine whether they are relevant and should be cited. There is no requirement to cite these works unless the editor has indicated otherwise.

Additional Editor Comments (if provided):

[Note: HTML markup is below. Please do not edit.]

Reviewers' comments:

Reviewer's Responses to Questions

**Comments to the Author**

1. Does this manuscript meet PLOS Global Public Health’s publication criteria?

Reviewer #1: Partly

**********

2. Has the statistical analysis been performed appropriately and rigorously?

Reviewer #1: No

**********

3. Have the authors made all data underlying the findings in their manuscript fully available (please refer to the Data Availability Statement at the start of the manuscript PDF file)?

The PLOS Data policy

Reviewer #1: Yes

**********

4. Is the manuscript presented in an intelligible fashion and written in standard English?

Reviewer #1: Yes

**********

Reviewer #1: This study addresses an important and timely topic and takes on a challenging methodological task by seeking to understand children’s experiences through parental perspectives. The authors provide a thoughtful contribution by exploring this perspective and by combining several methodological approaches. However, the manuscript would benefit from further clarification and refinement, particularly regarding the role and treatment of the questionnaire data.

See my comments to the manuscript in the attached document.

**********

what does this mean?). If published, this will include your full peer review and any attached files.

**Do you want your identity to be public for this peer review?**  If you choose “no”, your identity will remain anonymous but your review may still be made public.

For information about this choice, including consent withdrawal, please see our Privacy Policy

Reviewer #1: No

**********

[NOTE: If reviewer comments were submitted as an attachment file, they will be attached to this email and accessible via the submission site. Please log into your account, locate the manuscript record, and check for the action link "View Attachments". If this link does not appear, there are no attachment files.]

Attachment Submitted filename: PGPH-D-25-03876 Review comments.pdf

# Author comment: Author response to Decision Letter 1

**DOI:** 10.1371/journal.pgph.0005770.r002

27 Apr 2026

Attachment Submitted filename: Responses to Reviewers.docx

# Peer review: Decision Letter 1

**DOI:** 10.1371/journal.pgph.0005770.r003

**Authors:** Zulkarnain Jaafar

5 Aug 2026

PGPH-D-25-03876R1

Children’s participation in and access to free/low-cost physical activity opportunities in Northern Ireland (NI): parental perspectives.

PLOS Global Public Health

Dear Dr.McCafferty,

Thank you for submitting your manuscript to PLOS Global Public Health. After careful consideration, we feel that it has merit but does not fully meet PLOS Global Public Health’s publication criteria as it currently stands. Therefore, we invite you to submit a revised version of the manuscript that addresses the points raised during the review process.

==============================

EDITOR: Dear Author, please revise the manuscript based on the commnets provided by the reviewer.

==============================

Please submit your revised manuscript by 3 November 2026. If you will need more time than this to complete your revisions, please reply to this message or contact the journal office at globalpubhealth@plos.org. When you're ready to submit your revision, log on to https://www.editorialmanager.com/pgph/ and select the 'Submissions Needing Revision' folder to locate your manuscript file.

Please include the following items when submitting your revised manuscript:

- A letter that responds to each point raised by the editor and reviewer(s). You should upload this letter as a separate file labeled 'Response to Reviewers'.
- A marked-up copy of your manuscript that highlights changes made to the original version. You should upload this as a separate file labeled 'Revised Manuscript with Track Changes'.
- An unmarked version of your revised paper without tracked changes. You should upload this as a separate file labeled 'Manuscript'.

Guidelines for resubmitting your figure files are available below the reviewer comments at the end of this letter.

As the corresponding author, your ORCID iD is verified in the submission system and will appear in the published article. PLOS supports the use of ORCID, and we encourage all coauthors to register for an ORCID iD and use it as well. Please encourage your coauthors to verify their ORCID iD within the submission system before final acceptance, as unverified ORCID iDs will not appear in the published article. *Only*  the individual author can complete the verification step; PLOS staff *cannot*  verify ORCID iDs on behalf of authors.

We look forward to receiving your revised manuscript.

Kind regards,

Zulkarnain Jaafar

Academic Editor

PLOS Global Public Health

Journal Requirements:

If the reviewer comments include a recommendation to cite specific previously published works, please review and evaluate these publications to determine whether they are relevant and should be cited. There is no requirement to cite these works unless the editor has indicated otherwise.

Additional Editor Comments (if provided):

[Note: HTML markup is below. Please do not edit.]

Reviewers' comments:

Reviewer's Responses to Questions

**Comments to the Author**

Reviewer #2: (No Response)

**********

publication criteria?

Reviewer #2: Partly

**********

3. Has the statistical analysis been performed appropriately and rigorously?

Reviewer #2: Yes

**********

4. Have the authors made all data underlying the findings in their manuscript fully available (please refer to the Data Availability Statement at the start of the manuscript PDF file)?

The PLOS Data policy

Reviewer #2: Yes

**********

5. Is the manuscript presented in an intelligible fashion and written in standard English?

Reviewer #2: Yes

**********

Reviewer #2: I hope this letter finds you well. I had the opportunity to review your article titled, “Children’s participation in and access to free/low-cost physical activity opportunities in Northern Ireland (NI): the parent's perspective”, which was submitted the Plos Global Public Health.

Abstract

The abstract is strong in that it presents the research's purpose, background, methods, key findings, and policy implications relatively clearly. In particular, the critical perspective linking the "cost-of-living crisis" with children's participation in physical activity aligns well with current trends in international public health research. The study's objective is clear in that it explores parents' perceptions of "free/low-cost PA opportunities," and the concise presentation of the mixed-methods design (n=855 survey, n=29 interviews) allows readers to easily understand the study's scale and methodological structure. Furthermore, presenting key results—such as "29% increased participation in free activities" and "76% perceived insufficient opportunities"—along with numerical data enhances the effectiveness of the findings. The final sentence directly links to policy implications, giving it high practical relevance.

However, the abstracts generally remain at the level of descriptive summaries, and the theoretical contribution of the research is not sufficiently revealed. Since the current abstracts closely follow a structure of “problem exists → investigation → policy need,” they may feel somewhat lacking in novelty by the standards of Plos Global Public Health journals. The analysis is also presented primarily based on simple frequency results, appearing to lack analytical depth. In particular, the lack of a theoretical explanation regarding “why inequalities persist” in the abstract risks reading it as a simple policy report.

Introduction

The introduction generally has a stable logical flow and is relatively well-aligned with the style of international academic journals. Its strength lies in the fact that it first presents the health importance of physical activity for children and adolescents along with WHO guidelines, and then naturally connects this to the issues of low PA levels and socioeconomic inequality. In particular, the parallel use of NI regional data and global statistics secured both the regional context and international significance of the study. Furthermore, the connection between the recent socioeconomic phenomenon of the “cost-of-living crisis” and physical activity inequality is highly timely. Previous studies also extensively review SES disparity, green space inequality, leisure facility access, and subsidized PA intervention, so the literature coverage is relatively good.

However, the biggest weakness of the introduction is the absence of a clear theoretical framework. Currently, the introduction lists various prior studies, but there is no central theory to integrate them. For example, the academic completeness would have been much higher if the research problem had been structured using the socioecological model, the environmental justice framework, or health equity theory. Furthermore, the research gap is also somewhat weak. Although the phrase “evidence is limited” is repeated, there is a lack of explanation regarding the conceptual gap—specifically what existing research failed to explain. Consequently, while the introduction appears like a well-organized public health report, it lacks the strong theoretical positioning required by Plos Global Public Health.

Methods

The methodology section is generally very systematic and written with high reproducibility. It clearly explains the mixed-methods design, and it is good that the questionnaire and interview procedures are described separately. In particular, the clear description of ethical approval, inclusion criteria, recruitment strategy, and sample size calculation is positive in terms of methodological transparency. Additionally, the measurement of SES using deprivation tertiles aligns well with the research topic, and the measurement of parental support using the ACTS-MG scale is also valid in that it utilized an existing verification tool. Another strength of the qualitative analysis is that it sought to ensure the rigor of qualitative research by specifying the Braun & Clarke thematic analysis and the COREQ guideline.

On the other hand, quantitative analysis at the Plos Global Public Health level is somewhat simple. It is mostly centered on frequency analysis and group comparison, and lacks in-depth analyses such as regression, mediation, moderation, and multilevel analysis. It is regrettable that an explanatory model is absent, despite the research topic itself dealing with inequality and access. Furthermore, since all PA variables and access perception are based on self-reports, there is a high possibility of measurement bias. Another limitation is the exclusion of objective environmental data (GIS, facility audits, accelerometer data). Additionally, snowball sampling and social media recruitment methods can lead to representativeness issues, and a significant methodological limitation is that the sample is actually biased towards women, the highly educated, and the high-income bracket.

Results

The results section presents quantitative and qualitative results with a relatively clear distinction and includes a wealth of qualitative quotations. In particular, the connection of topics such as free/low-cost PA types, facility accessibility, swimming costs, rural transport issues, and school variability to specific participant quotes enhances the realism of the research. Furthermore, the mixed-methods integration, where quantitative results and qualitative narratives are connected complementarily, is relatively natural. For example, the issue of “lack of awareness” appears repeatedly in both surveys and interviews, reinforcing the consistency of the study.

However, the results remain at an overall descriptive level. Most of the outcomes are centered on frequency and perception, and there are few statistical models explaining the relationships between variables. In particular, the finding that “SES differences were not significant” could have been an interesting discovery, but further analysis on it is lacking. For example, the results would have been much stronger if interaction effects or subgroup analysis had been included. Additionally, while the qualitative findings are rich, the conceptual integration between themes is somewhat weak, and some results are presented repeatedly, which may feel a bit verbose. This reviewer considers it to be “interesting data but limited analytical depth.”

Discussion

The discussion clearly demonstrates an attempt to interpret the results by comparing them with existing literature. In particular, it is positive that the discussions on parental support, SES inequality, school role, and local government investment were connected to existing research. Furthermore, the suggestion that the cost-of-living crisis may have affected SES as a whole is relatively persuasive. The policy implications are also clear, clearly demonstrating practical implications such as local infrastructure investment, awareness promotion, and subsidy support. This is a strength of this study as an applied public health research.

However, the discussion also lacks theoretical contribution. Current discussions are mostly centered on "explanation of results + policy proposals" and lack broader conceptual implications. In particular, there is a lack of a deeper sociological interpretation regarding why free/low-cost opportunities do not sufficiently resolve inequality. Additionally, the latter part of the discussion is somewhat repetitive, and some sentences give the impression of simply repeating the results.

Conclusion

The conclusion is relatively concise yet effectively summarizes the core message of the study. In particular, it clearly emphasizes the lack of awareness, the need for long-term investment, and the issue of equitable access, and the connection of free/low-cost PA opportunities with a whole-system approach holds significant policy implications.

However, the conclusion remains somewhat predictable. While the message that “more support and accessibility are needed” is valid, stronger theoretical or empirical takeaways may be required by top-tier SSCI standards. Furthermore, the lack of a final message emphasizing the research's originality means that the new insights provided to the existing literature are conveyed relatively weakly.

**********

what does this mean?). If published, this will include your full peer review and any attached files.

**Do you want your identity to be public for this peer review?**  If you choose “no”, your identity will remain anonymous but your review may still be made public.

For information about this choice, including consent withdrawal, please see our Privacy Policy

Reviewer #2: No

**********

[NOTE: If reviewer comments were submitted as an attachment file, they will be attached to this email and accessible via the submission site. Please log into your account, locate the manuscript record, and check for the action link "View Attachments". If this link does not appear, there are no attachment files.]

# Author comment: Author response to Decision Letter 2

**DOI:** 10.1371/journal.pgph.0005770.r004

21 Sep 2026

Attachment Submitted filename: Response to reviewers .docx

# Peer review: Decision Letter 2

**DOI:** 10.1371/journal.pgph.0005770.r005

**Authors:** Zulkarnain Jaafar

15 Oct 2026

Free/low-cost physical activity opportunities for children and adolescents: parental perspectives on participation, access and provision.

PGPH-D-25-03876R2

Dear McCafferty,

We are pleased to inform you that your manuscript 'Free/low-cost physical activity opportunities for children and adolescents: parental perspectives on participation, access and provision.' has been provisionally accepted for publication in PLOS Global Public Health.

Before your manuscript can be formally accepted you will need to complete some formatting changes, which you will receive in a follow up email. A member of our team will be in touch with a set of requests.

Please note that your manuscript will not be scheduled for publication until you have made the required changes, so a swift response is appreciated.

IMPORTANT: The editorial review process is now complete. PLOS will only permit corrections to spelling, formatting or significant scientific errors from this point onwards. Requests for major changes, or any which affect the scientific understanding of your work, will cause delays to the publication date of your manuscript.

If your institution or institutions have a press office, please notify them about your upcoming paper to help maximize its impact. If they'll be preparing press materials, please inform our press team as soon as possible -- no later than 48 hours after receiving the formal acceptance. Your manuscript will remain under strict press embargo until 2 pm Eastern Time on the date of publication. For more information, please contact globalpubhealth@plos.org.

Thank you again for supporting Open Access publishing; we are looking forward to publishing your work in PLOS Global Public Health.

Best regards,

Zulkarnain Jaafar

Academic Editor

PLOS Global Public Health

***********************************************************

Reviewer Comments (if any, and for reference):

Reviewer's Responses to Questions

**Comments to the Author**

Reviewer #2: All comments have been addressed

**********

publication criteria?

Reviewer #2: Yes

**********

3. Has the statistical analysis been performed appropriately and rigorously?

Reviewer #2: Yes

**********

4. Have the authors made all data underlying the findings in their manuscript fully available (please refer to the Data Availability Statement at the start of the manuscript PDF file)?

The PLOS Data policy

Reviewer #2: No

**********

5. Is the manuscript presented in an intelligible fashion and written in standard English?

Reviewer #2: Yes

**********

Reviewer #2: Round 2

This study comprehensively analyzes—using both quantitative and qualitative approaches—the perceptions of parents and guardians regarding free or low-cost physical activity opportunities for children and adolescents; the topic is deemed highly timely and of significant practical value. In particular, by addressing issues of accessibility and participation in physical activity within the real-world context of rising living costs and socioeconomic disparities, the study provides meaningful insights for the fields of public health and the promotion of physical activity.

The primary strength of this study lies in its mixed-methods research design, which effectively combines quantitative and qualitative approaches. While a large-scale survey was used to identify overall participation patterns and variations based on socioeconomic characteristics, in-depth interviews with parents and guardians provided a detailed analysis of specific factors—such as geographic accessibility, transportation, facility quality, a lack of information, and program suitability—that are difficult to fully explain through survey data alone. Integrating the collection and analysis of both quantitative and qualitative data within a single study requires significant effort, yet this research successfully utilized the two approaches in a mutually complementary manner. This integration is considered a key strength that enhances the explanatory power and practical significance of the study's findings.

Furthermore, the research methods are clearly described throughout; regarding the quantitative analysis, the results are interpreted with caution, taking into account the characteristics of non-probability sampling and exploratory analysis. In the qualitative analysis, the thematic analysis procedures are detailed, multiple researchers participated in the analysis and interpretation, and the researchers' positionality was considered—demonstrating an effort to enhance the transparency of the research process and the reliability of the qualitative analysis. Indeed, this study examines the research questions from multiple perspectives by utilizing data from 855 survey respondents alongside in-depth interviews with 29 participants; this mixed-methods approach is considered a key methodological strength of the study.

Research findings consistently indicate that, while most parents and guardians reported their children’s participation in free or low-cost physical activities, many perceived local opportunities as insufficient. Qualitative findings complement these results by demonstrating that factors beyond cost—such as location, transportation, facility quality and suitability, and access to information—significantly influence actual participation. These results compellingly illustrate the need to consider actual availability and accessibility, rather than merely the provision of free or low-cost programs.

The revised manuscript provides a reasonably adequate explanation of the study's scope and limitations; notably, it explicitly acknowledges the limitations regarding representativeness inherent in non-probability sampling and exercises caution in interpretation to avoid overgeneralizing the findings to the population level. Furthermore, the qualitative analysis applies Braun and Clarke’s thematic analysis procedures and includes descriptions of the researcher's positionality and the analysis process, thereby demonstrating sufficient methodological transparency.

Overall, the authors appear to have thoroughly addressed the points raised during the previous review process, thereby enhancing the clarity and completeness of the manuscript. No significant issues affecting the validity of the study or its main conclusions were identified in the current version, and the research objectives, methods, results, and discussion are logically structured. In particular, the study is considered to hold significant academic and practical value for providing a broader and more in-depth understanding of children's and adolescents' participation in and access to free or low-cost physical activities through a mixed-methods approach combining quantitative and qualitative research.

Therefore, I judge that the currently revised manuscript has achieved the level of academic quality required for publication, and I recommend its publication.

**********

what does this mean?). If published, this will include your full peer review and any attached files.

**Do you want your identity to be public for this peer review?**  If you choose “no”, your identity will remain anonymous but your review may still be made public.

For information about this choice, including consent withdrawal, please see our Privacy Policy

Reviewer #2: No

**********
